# Impact of Pulmonary Ventilation Dysfunction on Prognosis of Patients with Coronary Artery Disease: A Single-Center, Observational Study

**DOI:** 10.31083/j.rcm2506197

**Published:** 2024-05-29

**Authors:** Yu-Shan Li, Qiang Ren, Jian Zhang, Yan-Chun Liang, Ya-Ling Han, Quan-Yu Zhang

**Affiliations:** ^1^State Key Laboratory of Frigid Zone Cardiovascular Diseases, Department of Cardiology, The General Hospital of Northern Theater Command, 110016 Shenyang, Liaoning, China; ^2^Postgraduate Training Base of The General Hospital of Northern Theater Command, Jinzhou Medical University, 110016 Shenyang, Liaoning, China; ^3^Department of Cardiology, Beifang Hospital of China Medical University, 110016 Shenyang, Liaoning, China

**Keywords:** coronary artery disease, pulmonary ventilation dysfunction, major adverse cardiovascular events, smoking

## Abstract

**Background:**

Patients with coronary artery disease (CAD) often experience pulmonary ventilation dysfunction following their initial event. However, there is insufficient research exploring the relationship between this dysfunction and CAD prognosis.

**Methods:**

To address this gap, a retrospective observational study was conducted involving 3800 CAD patients without prior pulmonary ventilation disease who underwent cardiopulmonary exercise testing (CPET) during hospitalization between November 2015 and September 2021. The primary endpoint was a composite of major adverse cardiovascular events (MACE), such as death, myocardial infarction (MI), repeat revascularization, and stroke. Propensity score matching (PSM) was used to minimize selection bias between the two groups, with a subgroup analysis stratified by smoking status.

**Results:**

The results showed that patients were divided into normal (n = 2159) and abnormal (n = 1641) groups based on their pulmonary ventilation function detected by CPET, with 1469 smokers and 2331 non-smokers. The median follow-up duration was 1237 (25–75% interquartile range 695–1596) days. The primary endpoint occurred in 390 patients (10.26%). 1472 patients in each of the two groups were enrolled in the current analysis after PSM, respectively. However, pulmonary function was not associated with MACE before (hazard ratio (HR) 1.20, 95% confidence interval (95% CI) 0.99–1.47; Log-rank *p* = 0.069) or after PSM (HR 1.07, 95% CI 0.86–1.34; Log-rank *p* = 0.545) among the entire population. Nonetheless, pulmonary ventilation dysfunction was significantly associated with an increased risk of MACE in smoking patients (HR 1.65, 95% CI 1.25–2.18; *p* < 0.001) but not in non-smoking patients (HR 0.81, 95% CI 0.60–1.09; *p* = 0.159). In addition, there was a significant interaction between current smoking status and pulmonary ventilation dysfunction on MACE (*p* for interaction < 0.001).

**Conclusions:**

Pulmonary ventilation dysfunction identified through CPET was independently associated with long-term poor prognosis in smoking patients with CAD but not in the overall population.

## 1. Introduction

Dyspnea is a common occurrence after index events in patients with coronary artery disease (CAD) [1, 2], and is associated with increased mortality [3, 4]. However, it is important to note that objective measures are necessary to confirm the presence of pulmonary ventilation dysfunction in cases of dyspnea. Cardiopulmonary exercise testing (CPET) is a common procedure for assessing the cardiopulmonary function of patients [5], which objectively identify symptoms such as dyspnea. However, the impact of pulmonary ventilation function on the prognosis of CAD patients is still an open issue.

Previous studies showed that cigarette smoke exposure is a risk factor result in the pulmonary dysfunction [6, 7]. Simultaneously, as an independent risk factor of CAD, smoking is associated with a poor prognosis of cardiovascular disease [8, 9]. However, the link of smoking, pulmonary dysfunction, and the prognosis of patients with CAD is unknown.

Therefore, we conducted an observational study to hypothesize that pulmonary ventilation dysfunction may play a significant role in major adverse cardiovascular events (MACE) in the CAD population. We also further explored whether smoking was associated with an increased likelihood of MACE in CAD patients with pulmonary ventilation dysfunction.

## 2. Materials and Methods

### 2.1 Study Design and Population

We performed a retrospective analysis of MACE within a cohort of CAD patients without lung disease, who received coronary angiography and post-procedure CPET during hospitalization between November 2015 and September 2021. Exclusion criteria were as follows: (i) combined with myocarditis, pericarditis, myocardiopathy, congenital heart disease, valvular heart disease or other structural heart diseases; (ii) with a history of pulmonary disease; (iii) mental impairment with limited ability to cooperate; (iv) unwillingness to participate or incomplete information. Categories of lung disease were defined based on forced expiratory volume in one second (${\mathrm{FEV}}_{1}$) and forced vital capacity (FVC), which can be measured during CPET in our cardiac rehabilitation center. Normal pulmonary ventilation function was defined as ${\mathrm{FEV}}_{1}$/FVC ≥70%. Abnormal pulmonary ventilation function was classified as obstructive (${\mathrm{FEV}}_{1}$/FVC <70% or <92% of predicted), restrictive (${\mathrm{FEV}}_{1}$/FVC ≥70% and FVC <80% of predicted), or mixed (${\mathrm{FEV}}_{1}$/FVC <70%) according to the American Thoracic Society/European Respiratory Society guidelines [10, 11, 12, 13]. Abnormal pulmonary ventilation function of any type diagnosed in the CPET report will be enrolled in the abnormal group, while normal pulmonary ventilation function will be enrolled in the normal group.

**Fig. 1.**
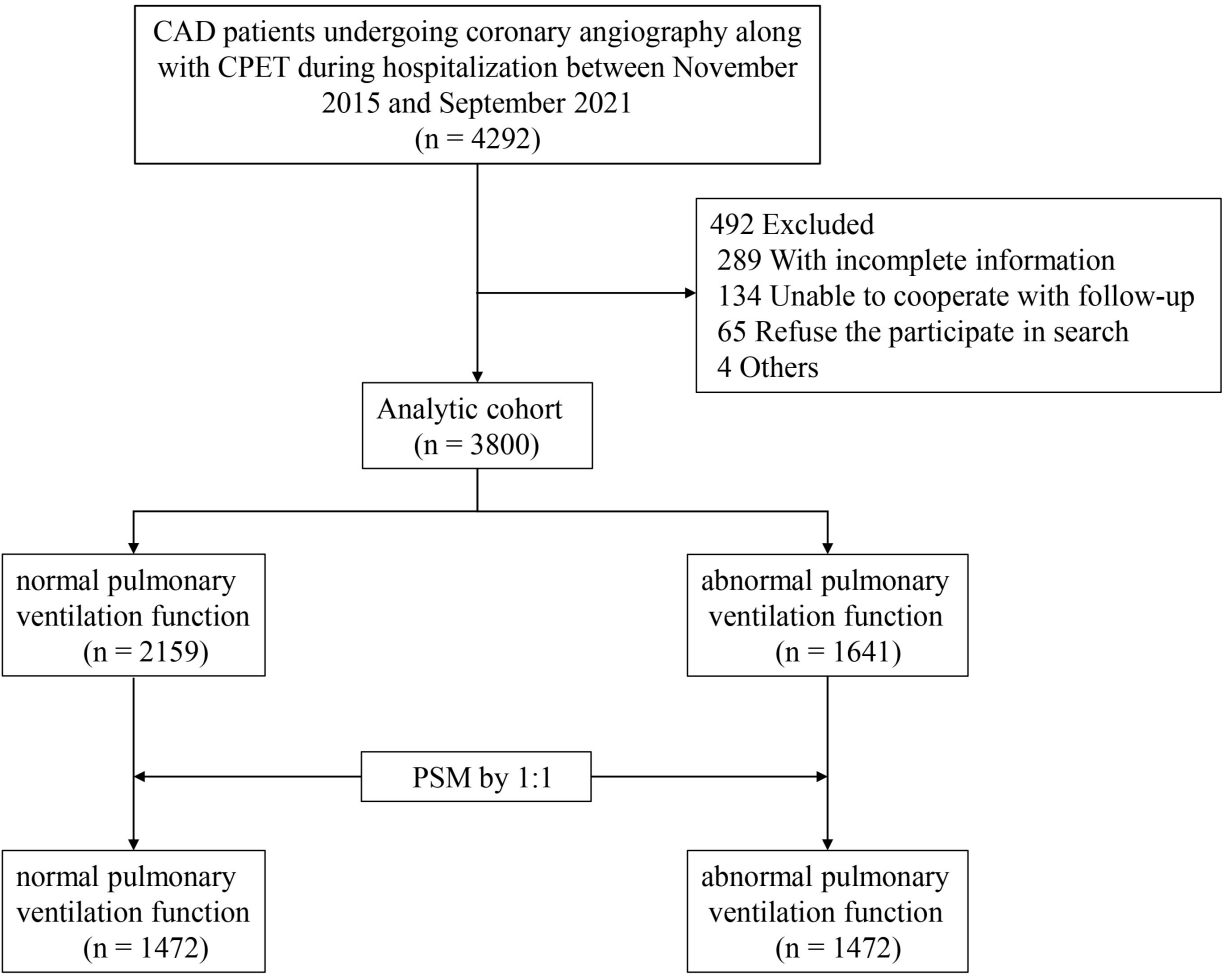
**Study Flow Diagram.** CAD, coronary artery disease; CPET, cardiopulmonary exercise testing; PSM, propensity score matching.

A total of 3800 patients were eligible for our study criterion, including 2159 normal pulmonary ventilation function CAD patients and 1641 abnormal pulmonary ventilation function CAD patients (Fig. 1). After performing propensity score matching (PSM), 1472 patients were included in each of the two groups for analysis. Additionally, to further investigate the effect of smoking status on MACE and pulmonary ventilation dysfunction, participants were divided into two groups based on smoking status, with 1469 smokers and 2331 non-smokers in the overall population. This study was approved by the Ethics Committee of General Hospital of Northern Theater Command, and an exemption for informed consent was approved simultaneously.

### 2.2 Cardiopulmonary Exercise Testing

CPET is a common method for assessing cardiovascular and pulmonary function in clinical practice, which has been routinely conducted in our center since 2015. Unlike general exercise and static lung function tests, CPET is an objective, quantitative, continuous, and non-invasive clinical detection method [14, 15].

Static pulmonary function parameters were measured in all patients and calibrated in a standardized manner using reference gases prior to each test. Following these measurements, dynamic pulmonary function indicators were measured using bicycle ergometers (SCHILLER, Baar, Switzerland) [16].

After a resting period of 1–2 minutes, a 2–3 minutes period of unloaded exercise was initiated at 60 revolutions per minute, followed by a continuous increase in load (gradually increased by 10% of the estimated exercise power based on patient age, height, and weight) until oxygen uptake and carbon dioxide excretion achieved equilibrium [17]. The exercise lasted until test termination according to the scientific statement from the American Heart Association. In the recovery period, patients exercised for approximately 2–3 minutes while rehabilitation technicians recorded CPET test results, including maximum kilogram oxygen uptake, metabolic equivalent, anaerobic threshold, and other indicators.

### 2.3 Clinical Data Collection and Follow-up

Patient information was obtained directly from the hospital information system, including demographic and clinical characteristics for all patients, past medical history, clinical diagnosis, medication at discharge, left ventricular ejection fraction (LVEF), procedural information, laboratory indicators, and CPET parameters. The follow-up was performed via telephone interview at 1, 6, 12, 36, and 60 months.

### 2.4 Study Endpoints

The primary endpoint was a composite of MACE within 60 months after discharge, including all-cause death, myocardial infarction (MI), repeat revascularization, and stroke. The secondary endpoint included the incidence of events for individual components of MACE, rehospitalization, the Bleeding Academic Research Consortium (BARC) defined type 2, 3 and 5 bleeding events, and all bleeding events.

Death and repeat revascularization were defined according to the Academic Research Consortium criteria [18], MI was defined according to the Fourth Universal Definition of Myocardial Infarction (2018) [19], and bleeding was defined according to the BARC criteria [20]. Stroke was defined as the sudden onset of vertigo, numbness, dysphasia, weakness, visual field defects, dysarthria, or other focal neurologic deficits due to vascular lesions of the brain such as hemorrhage, embolism, thrombosis, or a rupturing aneurysm that persists for >24 hours.

### 2.5 Statistical Analysis

Continuous variables were described as mean ± standard deviation (SD) for normally distributed measurement data and median ($P_{25}$–$P_{75}$) for non-normally distributed measurement data; categorical variables were described as frequencies with percentages. Continuous data were compared using the Student’s *t*-test and one-way analysis of variance (ANOVA), with correction for unequal variance when necessary. Chi-square tests or Fisher exact tests as appropriate for categorical variables.

To account for potential confounding attributable to such differences, a propensity score was used to match each patient in the abnormal group with a patient in the normal group with similar baseline characteristics, procedure information, and laboratory indicators. Greedy matching on propensity scores was performed with caliper of 0.2.

The Kaplan-Meier method was used to describe the cumulative incidence of endpoint events; comparisons were made with the Log-rank *t*-test. The association of smoking status with pulmonary ventilation function was evaluated using multivariable logistic regression analysis, and odds ratios (OR) with 95% confidence intervals (95% CI) were derived. The multivariate Cox regression analysis was performed specifically for smoking, and hazard ratios (HR) with 95% CI were derived. The interaction *p*-value was calculated to explore the interactive effect between smoking status and pulmonary ventilation dysfunction in the prognosis of CAD. A two-sided *p*-value < 0.05 was considered statistically significant. All statistical analyses were performed using R software (version 4.3.0; R Foundation for Statistical Computing, Vienna, Austria).

## 3. Results

### 3.1 Population Characteristics

A total of 3800 patients were finally included in this study, including 2159 normal and 1641 abnormal pulmonary ventilation function CAD patients. There are 261 (15.90%) patients with obstructive abnormalities, 1234 (75.20%) patients with restrictive abnormalities, and 149 (9.08%) patients with mixed abnormalities in abnormal group. Demographic characteristics, procedural information, laboratory indicators and CPET parameters of two groups before and after PSM were summarized in Tables 1,2,3. Before PSM, patients in abnormal group were older (58.49 ± 8.69 years *vs*. 56.32 ± 8.82 years, *p* < 0.001), more often male (80.32% *vs*. 74.20%, *p* < 0.001), more frequently smoking (41.38% *vs*. 36.59%, *p* = 0.003), more diabetes mellitus (29.92% *vs*. 25.01%, *p* < 0.001), had a higher systolic blood pressure (SBP) (137.66 ± 18.20 mmHg *vs*. 135.53 ± 18.38 mmHg, *p* < 0.001), had a lower LVEF (61.06 ± 5.98 *vs*. 61.70 ± 5.66, *p* < 0.001), had a higher SYNTAX score (the synergy between percutaneous coronary intervention with taxus and cardiac surgery score) [10.00 (6.00–15.50) *vs*. 10.00 (5.00–15.00), *p* = 0.010], had a higher N-terminal Pro-B-type natriuretic peptide (NT-PorBNP) [80.24 (36.75–207.70) pg/mL *vs*. 68.97 (32.77–174.15) pg/mL, *p* = 0.014] and lower high-density lipoprotein cholesterol (HDL-C) [1.05 (0.91–1.20) mmol/L *vs*. 1.08 (0.94–1.24) mmol/L, *p* < 0.001] compared with normal group.

**Table 1.** **Comparison of baseline characteristics before and after PSM in groups with normal and abnormal pulmonary ventilation function**.

Characteristic		Before PSM			After PSM		
		normal pulmonary ventilation function	abnormal pulmonary ventilation function	*p*-value	normal pulmonary ventilation function	abnormal pulmonary ventilation function	*p*-value
		(n = 2159)	(n = 1641)		(n = 1472)	(n = 1472)	
Age (years)		56.32 ± 8.82	58.49 ± 8.69	<0.001	57.74 ± 8.39	57.80 ± 8.62	0.847
Male, no. (%)		1602 (74.20)	1318 (80.32)	<0.001	1160 (78.80)	1159 (78.74)	>0.999
Current smoking, no. (%)		790 (36.59)	679 (41.38)	0.003	585 (39.74)	582 (39.54)	0.940
Current drinking, no. (%)		163 (7.55)	135 (8.23)	0.479	120 (8.15)	116 (7.88)	0.839
Past medical history, no. (%)							
	Hypertension	1272 (58.92)	1018 (62.04)	0.056	912 (61.96)	895 (60.80)	0.545
	DM	540 (25.01)	491 (29.92)	<0.001	404 (27.45)	423 (28.74)	0.460
	CKD	4 (0.19)	5 (0.30)	0.679	4 (0.27)	4 (0.27)	>0.999
	Previous PCI	654 (30.29)	491 (29.92)	0.833	428 (29.08)	453 (30.77)	0.334
	Previous stroke	194 (8.99)	176 (10.73)	0.082	151 (10.26)	141 (9.58)	0.579
	Previous MI	391 (18.11)	290 (17.67)	0.760	260 (17.66)	268 (18.21)	0.737
BMI (kg/$m^{2}$)		25.54 ± 2.89	25.45 ± 2.86	0.333	25.51 ± 2.91	25.47 ± 2.92	0.740
Heart rate (beats/min)		75.99 ± 11.77	76.22 ± 11.81	0.551	76.24 ± 11.71	76.08 ± 11.57	0.704
SBP (mmHg)		135.53 ± 18.38	137.66 ± 18.20	<0.001	136.99 ± 18.35	136.92 ± 17.84	0.920
DBP (mmHg)		81.04 ± 11.28	80.92 ± 11.46	0.754	81.17 ± 11.16	80.96 ± 11.31	0.615
LVEF (%)		61.70 ± 5.66	61.06 ± 5.98	<0.001	61.23 ± 5.87	61.30 ± 5.90	0.755
Diagnosis, no. (%)				0.551			0.975
	UA	1798 (83.28)	1345 (81.96)		1216 (82.61%)	1218 (82.74%)	
	NSTEMI	202 (9.36)	160 (9.75)		139 (9.44%)	143 (9.71%)	
	STEMI	156 (7.23)	135 (8.23)		116 (7.88%)	110 (7.47%)	
Medication at Discharge, no. (%)							
	Aspirin	2149 (99.54)	1626 (99.09)	0.133	1464 (99.46%)	1465 (99.52%)	>0.999
	Clopidogrel	1722 (79.76)	1316 (80.20)	0.771	1182 (80.30%)	1177 (79.96%)	0.853
	Ticagrelor	688 (31.87)	538 (32.78)	0.572	474 (32.20%)	475 (32.27%)	>0.999
	Statin	2131 (98.70)	1608 (97.99)	0.109	1449 (98.44%)	1449 (98.44%)	>0.999
	β-Blocker	1318 (61.05)	1020 (62.16)	0.507	920 (62.50%)	913 (62.02%)	0.820
	CCB	615 (28.49)	467 (28.46)	>0.999	421 (28.60%)	426 (28.94%)	0.871
	Nitrates	1383 (64.06)	1055 (64.29)	0.909	954 (64.81%)	943 (64.06%)	0.700
	PPI	996 (46.13)	766 (46.68)	0.763	691 (46.94%)	677 (45.99%)	0.660
	ACEI	521 (24.13)	417 (25.41)	0.385	367 (24.93%)	360 (24.46%)	0.798
	ARB	648 (30.01)	503 (30.65)	0.698	457 (31.05%)	445 (30.23%)	>0.999
	Diuretic	77 (3.57)	71 (4.33)	0.265	58 (3.94%)	56 (3.80%)	0.924

Note: PSM, propensity score matching; DM, diabetes mellitus; CKD, chronic kidney disease; PCI, percutaneous coronary intervention; MI, myocardial infarction; BMI, body mass index; SBP, systolic blood pressure; DBP, diastolic blood pressure; LVEF, left ventricular ejection fraction; UA, unstable angina; NSTEMI, Non-ST segment elevation myocardial infarction; STEMI, ST segment elevation myocardial infarction; CCB, calcium channel blockers; PPI, proton-pump inhibitor; ACEI, angiotensin converting enzyme inhibitors; ARB, angiotensin receptor blocker.

**Table 2.** **Comparison of procedural information and laboratory indicators before and after PSM in groups with normal and abnormal pulmonary ventilation function**.

Characteristic		Before PSM			After PSM		
		normal pulmonary ventilation function	abnormal pulmonary ventilation function	*p*-value	normal pulmonary ventilation function	abnormal pulmonary ventilation function	*p*-value
		(n = 2159)	(n = 1641)		(n = 1472)	(n = 1472)	
Procedural information							
	SYNTAX score	10.00 (5.00–15.00)	10.00 (6.00–15.50)	0.013	10.00 (5.00–15.00)	10.00 (6.00–15.00)	0.683
	Three-vessel disease, no. (%)	340 (15.75)	260 (15.84)	0.972	238 (16.17%)	229 (15.56%)	0.687
	PCI, no. (%)	1567 (72.58)	1218 (74.22)	0.273	1091 (74.12%)	1084 (73.64%)	0.801
	CTO, no. (%)	70 (3.24)	55 (3.35)	0.924	49 (3.33%)	50 (3.40%)	>0.999
Laboratory parameters							
	Hemoglobin (g/L)	140.50 ± 13.24	140.01 ± 13.91	0.269	140.24 ± 12.99	140.15 ± 13.94	0.850
	NT-PorBNP (pg/mL)	68.97 (32.77–174.15)	80.24 (36.75–207.70)	0.014	73.91 (35.02–188.00)	77.65 (35.37–190.23)	0.725
	Total cholesterol (mmol/L)	3.69 (3.13–4.50)	3.72 (3.10–4.45)	0.788	3.68 (3.10–4.47)	3.71 (3.09–4.44)	0.666
	Triglycerides (mmol/L)	1.36 (0.97–1.93)	1.42 (1.02–1.98)	0.053	1.39 (0.98–1.96)	1.41 (1.02–1.97)	0.802
	LDL-C (mmol/L)	1.99 (1.57–2.56)	1.99 (1.58–2.55)	0.902	1.99 (1.57–2.54)	1.98 (1.57–2.53)	0.688
	HDL-C (mmol/L)	1.08 (0.94–1.24)	1.05 (0.91–1.20)	<0.001	1.05 (0.92–1.21)	1.05 (0.91–1.21)	0.847
	Troponin (ng/mL)	0.01 (0.01–0.02)	0.01 (0.01–0.02)	0.637	0.01 (0.01–0.02)	0.01 (0.01–0.02)	0.645
	Scr (umol/L)	68.98 (59.61–78.60)	69.58 (60.09–79.12)	0.244	69.52 (60.46–79.20)	69.40 (59.61–78.81)	0.712
	ALT (U/L)	22.64 (16.00–33.00)	22.30 (16.00–34.00)	0.628	22.55 (15.91–33.00)	22.72 (16.00–34.00)	0.975
	CKMB (U/L)	13.00 (10.00–16.00)	12.00 (10.00–16.00)	0.364	12.40 (10.00–15.23)	12.00 (10.00–16.00)	0.538

Note: PSM, propensity score matching; SYNTAX score, the synergy between percutaneous coronary intervention with taxus and cardiac surgery score; PCI, percutaneous coronary intervention; CTO, chronic total occlusion; NT-PorBNP, N-terminal Pro-B-type natriuretic peptide; LDL-C, low-density lipoprotein cholesterol; HDL-C, high-density lipoprotein cholesterol; Scr, serum creatinine; ALT, alanine aminotransferase; CKMB, creatine kinase MB.

**Table 3.** **Comparison CPET parameters before and after PSM in groups with normal and abnormal pulmonary ventilation function**.

Characteristic		Before PSM			After PSM		
		normal pulmonary ventilation function	abnormal pulmonary ventilation function	*p*-value	normal pulmonary ventilation function	abnormal pulmonary ventilation function	*p*-value
		(n = 2159)	(n = 1641)		(n = 1472)	(n = 1472)	
Static lung function							
	FVC (L/min)	3.38 ± 0.73	2.76 ± 0.66	<0.001	3.40 ± 0.70	2.77 ± 0.67	<0.001
	FVC (% predicted)	87.65 ± 5.59	72.68 ± 10.46	<0.001	87.67 ± 5.62	72.89 ± 10.40	<0.001
	${\mathrm{FEV}}_{1}$ (L)	2.78 ± 0.62	2.16 ± 0.53	<0.001	2.78 ± 0.59	2.17 ± 0.54	<0.001
	${\mathrm{FEV}}_{1}$ (% predicted)	87.71 ± 6.67	71.29 ± 11.75	<0.001	87.61 ± 6.73	71.39 ± 11.68	<0.001
	${\mathrm{FEV}}_{1}$/FVC (%)	82.14 ± 6.26	79.30 ± 14.30	<0.001	81.71 ± 6.19	79.34 ± 14.10	<0.001
	${\mathrm{FEV}}_{1}$/FVC (% predicted)	81.87 ± 8.27	82.19 ± 8.24	0.24	81.99 ± 8.32	82.29 ± 8.21	0.316
	VCmax (L/min)	3.53 ± 0.75	2.99 ± 0.66	<0.001	3.55 ± 0.73	3.00 ± 0.67	<0.001
	MVV (L/min)	111.69 ± 29.64	94.29 ± 26.14	<0.001	111.21 ± 28.56	94.89 ± 26.31	<0.001
Dynamic lung function							
	Duration (seconds)	408.32 ± 113.52	389.12 ± 106.82	<0.001	407.58 ± 111.62	390.28 ± 107.45	<0.001
	Peak heart rate (time/min)	120.66 ± 19.48	116.85 ± 18.64	<0.001	119.17 ± 18.74	117.29 ± 18.73	0.007
	Peak workload (W)	96.98 ± 29.99	92.00 ± 27.94	<0.001	97.00 ± 29.48	92.38 ± 28.23	<0.001
	Peak MET	4.63 ± 1.16	4.26 ± 1.06	<0.001	4.60 ± 1.14	4.29 ± 1.07	<0.001
	MET AT	3.20 ± 0.62	3.03 ± 0.58	<0.001	3.20 ± 0.62	3.04 ± 0.59	<0.001
	Peak ${\mathrm{VO}}_{2}$ (mL/kg/min)	16.20 ± 4.05	14.92 ± 3.72	<0.001	16.11 ± 3.99	15.01 ± 3.73	<0.001
	${\mathrm{VO}}_{2}$ AT (mL/kg/min)	11.18 ± 2.16	10.60 ± 2.05	<0.001	11.19 ± 2.17	10.62 ± 2.06	<0.001
	Peak VE (L/min)	40.13 ± 12.41	38.29 ± 11.59	<0.001	40.29 ± 12.11	38.32 ± 11.68	<0.001
	Peak oxygen pulse (mL/beat)	9.89 ± 2.54	9.47 ± 2.42	<0.001	10.01 ± 2.51	9.49 ± 2.45	<0.001
	Maximal respiratory rate (time/min)	28.46 ± 5.57	29.74 ± 5.64	<0.001	28.31 ± 5.50	29.73 ± 5.67	<0.001
	VE/${\mathrm{VO}}_{2}$	28.73 ± 3.52	29.70 ± 4.08	<0.001	28.96 ± 3.55	29.50 ± 4.04	<0.001
	VE/${\mathrm{VCO}}_{2}$	29.66 ± 3.43	30.68 ± 3.93	<0.001	29.85 ± 3.45	30.47 ± 3.88	<0.001
	HRR	42.53 ± 19.55	44.14 ± 18.49	0.010	42.61 ± 18.98	44.41 ± 18.58	0.009
	BR (%)	63.06 ± 10.16	57.67 ± 13.15	<0.001	62.71 ± 10.35	57.89 ± 13.10	<0.001

Note: CPET, cardiopulmonary exercise testing; PSM, propensity score matching; FVC, forced vital capacity; ${\mathrm{FEV}}_{1}$, forced expiratory volume in one second; VCmax, maximum vital capacity; MVV, maximal voluntary ventilation; MET, metabolic equivalent; AT, anaerobic threshold; ${\mathrm{VO}}_{2}$, oxygen consumption; VE, minute ventilation volume; VE/${\mathrm{VO}}_{2}$, the minute ventilation-oxygen dioxide production; VE/${\mathrm{VCO}}_{2}$, the minute ventilation-carbon dioxide production; HRR, heart rate reserve; BR, breathing reserve.

1472 patients in each of the two groups were enrolled in the current analysis after PSM, respectively. Baseline population characteristics, procedural information and laboratory indicators characteristics were similar in the normal and abnormal cohorts after PSM (all *p* > 0.05, Tables 1,2). Patients in abnormal group had a higher ${\mathrm{FEV}}_{1}$/FVC of predicted before (82.19 ± 8.24 *vs*. 81.87 ± 8.27, *p* = 0.240) and after PSM (82.29 ± 8.21 *vs*. 81.99 ± 8.32, *p* = 0.316). There were significant differences of other CPET parameters between normal and abnormal cohorts before and after PSM (all *p* < 0.05, Table 3).

### 3.2 Primary Endpoint

**Fig. 2.**
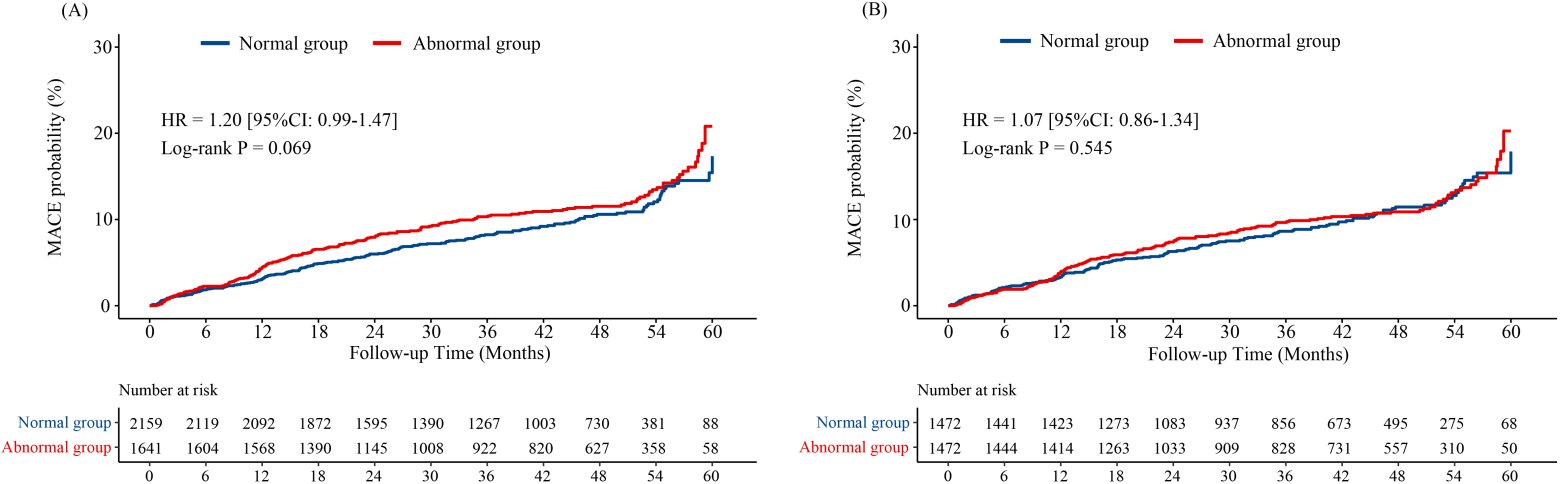
**Kaplan-Meier curves for MACE probability through 5-year follow-up.** (A) Before PSM. (B) After PSM. MACE, major adverse cardiovascular events; PSM, propensity score matching; HR, hazard ratio; 95% CI, 95% confidence interval.

During a median follow-up duration of 1237 days (25–75% interquartile range 695–1596 days), the primary endpoint, MACE, occurred in 390 (10.26%) patients. There was no significant difference in the incidence of MACE between the two groups, both of before and after PSM (before PSM: *p* = 0.051; after PSM: *p* = 0.952; Table 4). The Kaplan-Meier analysis also showed there was no significant difference between pulmonary ventilation dysfunction and MACE, before (HR 1.20, 95% CI 0.99–1.47; Log-rank *p* = 0.069; Fig. 2A) or after PSM (HR 1.07, 95% CI 0.86–1.34; Log-rank *p* = 0.545; Fig. 2B).

**Table 4.** **Clinical outcomes before and after PSM in groups with normal and abnormal pulmonary ventilation function**.

Characteristic		Before PSM			After PSM		
		normal pulmonary ventilation function	abnormal pulmonary ventilation function	*p*-value	normal pulmonary ventilation function	abnormal pulmonary ventilation function	*p*-value
		(n = 2159)	(n = 1641)		(n = 1472)	(n = 1472)	
MACE, no. (%)		203 (9.40)	187 (11.40)	0.051	147 (9.99)	158 (10.73)	0.545
	Death	16 (0.74)	28 (1.71)	0.009	12 (0.82)	19 (1.29)	0.279
	MI	19 (0.88)	21 (1.28)	0.301	14 (0.95)	19 (1.29)	0.484
	Repeated revascularization	161 (7.46)	142 (8.65)	0.198	113 (7.68)	127 (8.63)	0.381
	Stroke	21 (0.97)	19 (1.16)	0.694	17 (1.15)	15 (1.02)	0.859
Bleeding, no. (%)		419 (19.41)	299 (18.22)	0.377	288 (19.57)	277 (18.82)	0.640
	BARC 2, 3 and 5	45 (2.08)	40 (2.44)	0.536	31 (2.11)	38 (2.58)	0.465
Rehospitalization, no. (%)		463 (21.45)	387 (23.58)	0.127	325 (22.08)	345 (23.44)	0.404

Note: PSM, propensity score matching; MACE, major adverse cardiovascular events, including all-cause deaths, MI, strokes, and repeated revascularizations; MI, myocardial infarction; BARC, bleeding academic research consortium.

**Fig. 3.**
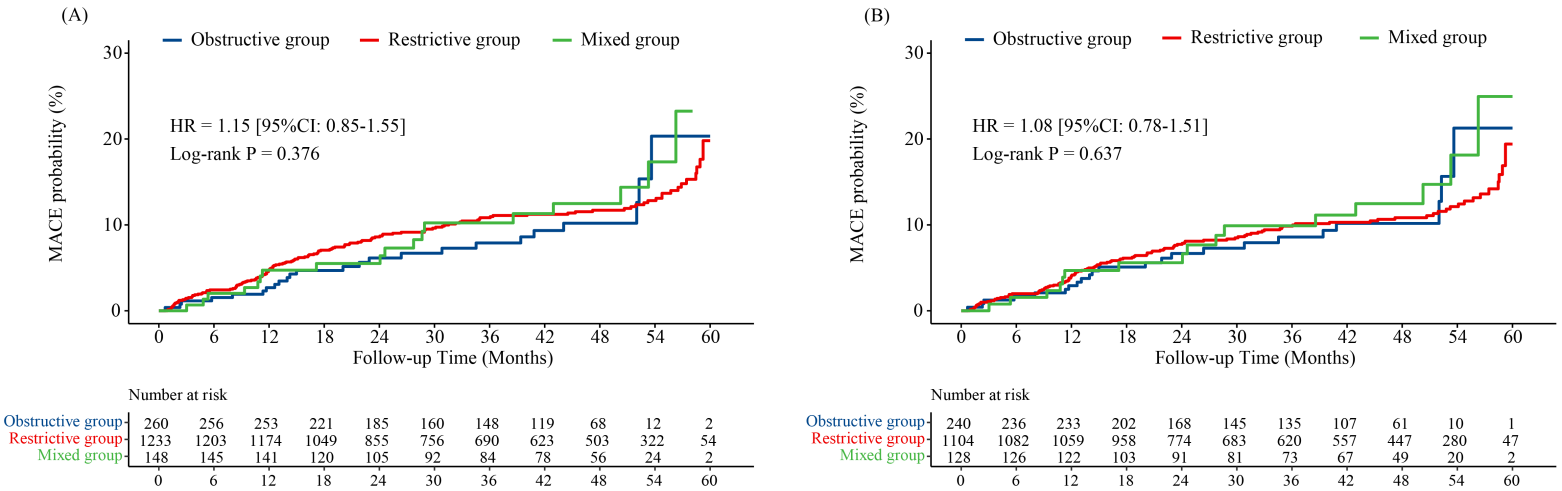
**Kaplan-Meier curves for MACE probability in different type of abnormal pulmonary ventilation function through 5-year follow-up.** (A) Before PSM. (B) After PSM. MACE, major adverse cardiovascular events; PSM, propensity score matching; HR, hazard ratio; 95% CI, 95% confidence interval.

There was no significant difference in the incidence of MACE in different type of abnormal pulmonary ventilation function, both of before and after PSM (before PSM: *p* = 0.450; after PSM: *p* = 0.690; Table 5). The Kaplan-Meier analysis also showed there was no significant in different type of abnormal pulmonary ventilation function and MACE, before (HR 1.15, 95% CI 0.85–1.55; Log-rank *p* = 0.376; Fig. 3A) or after PSM (HR 1.08, 95% CI 0.78–1.51; Log-rank *p* = 0.637; Fig. 3B). Demographic characteristics, procedural information, laboratory indicators and CPET parameters of three groups before and after PSM were summarized in **Supplementary Tables 1–3**.

**Table 5.** **Clinical outcomes before and after PSM in different type of abnormal pulmonary ventilation function**.

Characteristic		Before PSM				After PSM			
		Mixed pulmonary ventilation dysfunction	Obstructive pulmonary ventilation dysfunction	Restricted pulmonary ventilation dysfunction	*p*-value	Mixed pulmonary ventilation dysfunction	Obstructive pulmonary ventilation dysfunction	Restricted pulmonary ventilation dysfunction	*p*-value
		(n = 148)	(n = 260)	(n = 1233)		(n = 128)	(n = 240)	(n = 1104)	
MACE, no. (%)		19 (12.84)	24 (9.23)	144 (11.68)	0.450	16 (12.50)	23 (9.58)	119 (10.78)	0.690
	Death	2 (1.35)	2 (0.77)	24 (1.95)	0.390	1 (0.78)	2 (0.83)	16 (1.45)	0.650
	MI	2 (1.35)	2 (0.77)	17 (1.38)	0.730	2 (1.56)	1 (0.42)	16 (1.45)	0.420
	Repeated revascularization	15 (10.14)	21 (8.08)	106 (8.60)	0.770	14 (10.94)	20 (8.33)	93 (8.42)	0.620
	Stroke	2 (1.35)	2 (0.77)	15 (1.22)	0.810	1 (0.78)	2 (0.83)	12 (1.09)	0.900
Bleeding, no. (%)		29 (19.59)	47 (18.08)	223 (18.09)	0.900	25 (19.53)	44 (18.33)	208 (18.84)	0.960
	BARC 2, 3 and 5	3 (2.03)	4 (1.54)	33 (2.68)	0.530	3 (2.34)	4 (1.67)	31 (2.81)	0.590
Rehospitalization, no. (%)		38 (25.68)	62 (23.85)	287 (23.28)	0.800	32 (25.00)	57 (23.75)	256 (23.19)	0.890

Note: PSM, propensity score matching; MACE, major adverse cardiovascular events; MI, myocardial infarction; BARC, bleeding academic research consortium.

### 3.3 Secondary Endpoints

Patients in the abnormal group had a significantly higher rate of all-cause death compared with normal group before PSM (1.71% *vs*. 0.74%, *p* = 0.009, Table 4). After PSM, however, no significant difference was found in respect to the rate of all-cause death between the two groups (all *p* > 0.05). The Kaplan-Meier analysis also showed that, significant association was observed between two groups before PSM (HR 2.25, 95% CI 1.21–4.15; Log-rank *p* = 0.010; **Supplementary Fig. 1A**), but not after PSM (HR 1.54, 95% CI 0.75–3.17; Log-rank *p* = 0.243; **Supplementary Fig. 1B**). The rates of other secondary endpoints were also similar between the two groups before or after PSM (**Supplementary Figs. 2–7**).

### 3.4 Association of Smoking with Pulmonary Ventilation Dysfunction

**Fig. 4.**
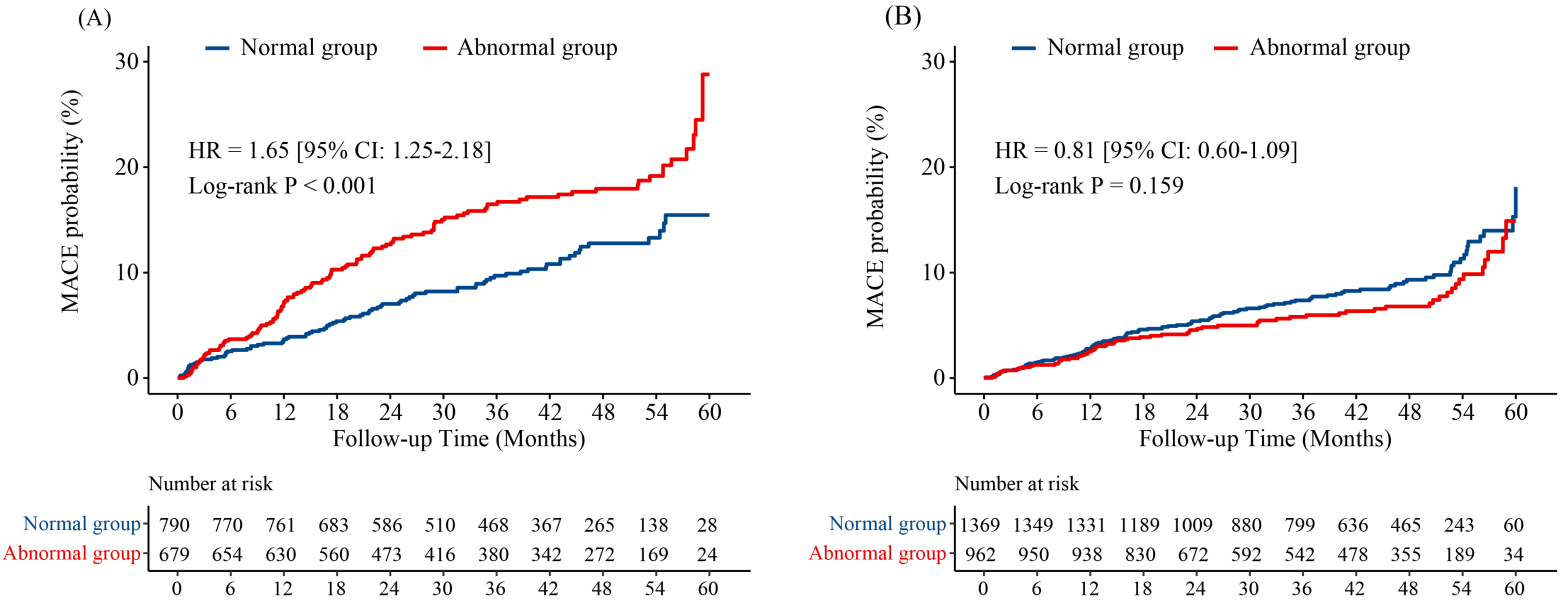
**Kaplan-Meier curves for MACE probability through 5-year follow-up.** (A) Smoking. (B) Non-smoking. MACE, major adverse cardiovascular events; HR, hazard ratio; 95% CI, 95% confidence interval.

The present study firstly conducted a multiple logistic regression analysis to determine the predictor of the occurrence of pulmonary dysfunction (**Supplementary Table 4**). After adjustment for age (OR 1.04, 95% CI 1.03–1.04; *p* < 0.001), male (OR 1.43, 95% CI 1.20–1.71; *p* < 0.001), diabetes mellitus (DM) (OR 1.20, 95% CI 1.04–1.40; *p* = 0.014), SBP (OR 1.00, 95% CI 1.00–1.01; *p* = 0.017), LVEF (OR 0.98, 95% CI 0.97–0.99; *p* = 0.003), and HDL-C (OR 0.57, 95% CI 0.43–0.76; *p* < 0.001), smoking was independently associated with the occurrence of pulmonary ventilation dysfunction among CAD patients (OR 1.20, 95% CI 1.04–1.38; *p* = 0.015). Furthermore, we performed the subgroup analysis stratified by smoking status in respect to MACE. We found that, pulmonary ventilation dysfunction was independently and significantly associated with an increased risk of MACE among smoking patients (HR 1.65, 95% CI 1.25–2.18; *p* < 0.001; Fig. 4A), but not in non–smoking patients (HR 0.81, 95% CI 0.60–1.09; *p* = 0.159; Fig. 4B). The multivariate Cox analysis specifically looking at current smoking showed that pulmonary ventilation dysfunction (HR 1.42, 95% CI 1.07–1.89; *p* = 0.015), current drinking (HR 1.77, 95% CI 1.10–2.85; *p* = 0.030), previous percutaneous coronary intervention (PCI) (HR 1.67, 95% CI 1.24–2.24; *p* < 0.001), creatine kinase MB (CKMB) (HR 1.00, 95% CI 1.00–1.01; *p* = 0.016), the minute ventilation-carbon dioxide production (VE/${\mathrm{VCO}}_{2}$) (HR 1.07, 95% CI 1.03–1.11; *p* < 0.001), SYNTAX score (HR 1.04, 95% CI 1.03–1.06; *p* < 0.001) were risk factors of MACE (Table 6). In addition, there was a significant interaction between current smoking status and pulmonary ventilation dysfunction on MACE (*p* for interaction < 0.001, Table 7).

**Table 6.** **The multivariate Cox regression analysis specifically looking at current smoking**.

Characteristic	Univariate Cox regression analysis			Multivariate Cox regression analysis		
	HR	95% CI	*p*-value	HR	95% CI	*p*-value
Pulmonary ventilation dysfunction	1.65	1.25–2.18	<0.001	1.42	1.07–1.89	0.015
Current drinking	1.94	1.21–3.11	0.012	1.77	1.10–2.85	0.030
Previous PCI	1.59	1.19–2.12	0.002	1.67	1.24–2.24	<0.001
CKMB	1.00	1.00–1.01	0.023	1.00	1.00–1.01	0.016
VE/${\mathrm{VCO}}_{2}$	1.09	1.05–1.13	<0.001	1.07	1.03–1.11	<0.001
SYNTAX score	1.04	1.03–1.06	<0.001	1.04	1.03–1.06	<0.001

Note: PCI, percutaneous coronary intervention; CKMB, creatine kinase MB; VE/${\mathrm{VO}}_{2}$, the minute ventilation-oxygen dioxide production; SYNTAX score, the synergy between percutaneous coronary intervention with taxus and cardiac surgery score; HR, hazard ratio; 95% CI, 95% confidence interval.

**Table 7.** **Interactive effect of smoking status and pulmonary ventilation function**.

Characteristic	Pulmonary ventilation function	*p*-value for interaction
Smoking status		
Current smoking	HR 1.65, 95% CI 1.25–2.18; *p* < 0.001	<0.001
Non-smoking	HR 0.81, 95% CI 0.60–1.09; *p* = 0.159	

HR, hazard ratio; 95% CI, 95% confidence interval.

## 4. Discussion

In our daily work, patients were often anxiety about abnormal pulmonary ventilation function in CPET reports. Meanwhile, the association between pulmonary ventilation dysfunction and CAD prognosis was inconclusive. Thus, we performed the present study, which firstly examined the association between pulmonary ventilation dysfunction measured by CPET and the prognosis of CAD. Present data showed that: (i) there was no difference in the incidence of MACE in CAD stratified by pulmonary ventilation function; (ii) before PSM, a higher mortality was found in patients with pulmonary ventilation dysfunction, while there was no difference after PSM; (iii) among the smoking patients, pulmonary ventilation dysfunction was an independent risk factor of MACE.

Dyspnea is a subjective symptom of CAD patients, but the existence of pulmonary ventilation dysfunction is uncertain. Parameters of pulmonary ventilation function measured by CPET provide more objective and reliable results than self-reported symptoms. The present study, which analyzed a cohort of 3800 CAD patients undergoing CPET, demonstrated that the impact of MACE was similar among patients with normal or abnormal pulmonary ventilation function. This is consistent with the findings of the CONFIRM registry study, which reported that the presence of dyspnea did not increase the long-term risk for MACE in obstructive CAD [21]. However, other studies have reported conflicting results, indicating that dyspnea is associated with worse clinical outcomes among CAD patients [21, 22, 23, 24]. This phenomenon may be due to enrollment variety in different studies. The present study included patients without history of pulmonary dysfunction, but contradictory studies enrolled patients with pulmonary symptoms such as dyspnea [21, 22, 23, 24, 25] who may already had pulmonary ventilation dysfunction before had symptoms of CAD. As numerous known, the presence of pulmonary dysfunction prior to the occurrence of CAD may impact the clinical outcomes.

Consistent with previous research [26, 27], the present study showed that the overall all-cause mortality was higher in patients with pulmonary ventilation dysfunction before PSM. However, after adjustment by PSM, the difference was not significant. Founded on the present findings, considering that some patients with pulmonary ventilation dysfunction may be transient or compensatory in some patients, leading to pulmonary ventilation dysfunction had no impact on the prognosis of CAD patients. Moreover, pulmonary ventilation dysfunction determined before discharge had limited value of predicting long-term prognosis of CAD patients. Therefore, the periodic inspection of pulmonary ventilation function may be required for CAD patients, which confirm whether pulmonary ventilation dysfunction still exists and identify the impact on long-term prognosis of CAD. Previous studies have found that dyspnea is commonly associated with ticagrelor therapy [28, 29, 30]. In most previous studies, the subjective feelings of patients with dyspnea had been used as an endpoint. However, patients with pulmonary ventilation dysfunction may not have symptom of dyspnea. In the present study, 30% of both groups were on ticagrelor, but there was no difference. Therefore, pulmonary dysfunction was not associated with ticagrelor therapy.

Smoking is a risk factor to the progression and adverse prognosis of cardiovascular and pulmonary disease [31, 32]. The present data demonstrated that pulmonary ventilation dysfunction is an independent risk factor for MACE in smoking patients. A study of 3103 smoking patients with ischemic heart disease also reported similar results [33]. However, in non-smoking participants, there was no significant correlation between pulmonary ventilation dysfunction and MACE, which is consistent with the overall study population. Previous studies have confirmed that cigarette smoke exposure has been associated with vasodilator dysfunction and atherosclerotic pathological changes, which impaired blood flow reconstruction and deteriorated the prognosis of patients with coronary artery disease [9]. Additionally, cigarette smoke exposure disrupted the epithelial barrier, leading to the development of upper respiratory tract infections that may further exacerbate dyspnea [6]. The present data also showed the interaction between current smoking status and pulmonary ventilation dysfunction. Pathological changes in the lungs caused by smoking may deteriorate the prognosis of CAD patients, compared with non-smoking patients. In considering the results of this study, pulmonary ventilation function may be an important factor in evaluating the outcomes of smoking patients with CAD.

There are some limitations of the present study which should be considered. First, this study was a single-center retrospective analysis, with possible selection bias, despite PSM was conducted. The prospective multicenter study is needed to further support the conclusions. Second, pulmonary ventilation function might be relative to the prognosis of CAD, while no pulmonary ventilation function endpoints were followed up in present study. Although the mortality of the group with abnormal pulmonary ventilation function was nearly twice that of the normal group, there was no statistically significant difference. That may be due to insufficient sample size. A larger sample size may reveal differences in further analysis. Third, the CPET is unable to assess the severity of the prognosis of pulmonary ventilation function. Therefore, the detailed clinical assessment by the respiratory physician may be better. Fourth, only the hard endpoints such as MACE and all-cause death were analyzed in this study. Functional status and quality of life were not evaluated due to the insufficiency of follow-up. We intend to focus on this issue in another prospective observational study. Finally, our analysis included smoking status as a subgroup analysis to investigate the role of pulmonary ventilation dysfunction on MACE in CAD patients, which need further research to validate.

## 5. Conclusions

No association was observed between pulmonary ventilation dysfunction detected by CPET and long-term MACE in the entire population of patients with CAD. However, in the subgroup analysis including smoking patients, pulmonary ventilation dysfunction may be an independent risk factor of poor prognosis of CAD.

## Data Availability

The data sets analyzed during the current study are not publicly available due to restrictions apply to the availability of these data but are available from the corresponding author on reasonable request.

## Abbreviations

CAD, coronary artery disease; CPET, cardiopulmonary exercise testing; MACE, major adverse cardiovascular events; ${\mathrm{FEV}}_{1}$, forced expiratory volume in one second; FVC, forced vital capacity; PSM, propensity score match; LVEF, left ventricular ejection fraction; MI, myocardial infarction; BARC, Bleeding Academic Research Consortium; DM, diabetes mellitus; CKD, chronic kidney disease; PCI, percutaneous coronary intervention; MI, myocardial infarction; BMI, body mass index; SBP, systolic blood pressure; DBP, diastolic blood pressure; UA, unstable angina; NSTEMI, Non-ST segment elevation myocardial infarction; STEMI, ST segment elevation myocardial infarction; CCB, calcium channel blockers; PPI, proton-pump inhibitor; ACEI, angiotensin converting enzyme inhibitors; ARB, angiotensin receptor blocker; SYNTAX score, the synergy between percutaneous coronary intervention with taxus and cardiac surgery score; CTO, chronic total occlusion; NT-PorBNP, N-terminal Pro-B-type natriuretic peptide; LDL-C, low-density lipoprotein cholesterol; HDL-C, high-density lipoprotein cholesterol; Scr, serum creatinine; ALT, alanine aminotransferase; CKMB, creatine kinase MB; VCmax, maximum vital capacity; MVV, maximal voluntary ventilation; MET, metabolic equivalent; AT, anaerobic threshold; ${\mathrm{VO}}_{2}$, oxygen consumption; VE, minute ventilation volume; VE/${\mathrm{VO}}_{2}$, the minute ventilation-oxygen dioxide production; VE/${\mathrm{VCO}}_{2}$, the minute ventilation-carbon dioxide production; HRR, heart rate reserve; BR, breathing reserve.

## Supplementary Material

Supplementary material associated with this article can be found, in the online version, at https://doi.org/10.31083/j.rcm2506197.
