# Supplementary material for: Impact of Pulmonary Ventilation Dysfunction on Prognosis of Patients with Coronary Artery Disease: A Single-Center, Observational Study
Source: Rev Cardiovasc Med. 2024 May 29;25(6):197. doi: 10.31083/j.rcm2506197 (PMC11270079; doi:10.31083/j.rcm2506197)
Supplement: Supplementary file 1 [file 2153-8174-25-6-197-s1.docx]

| **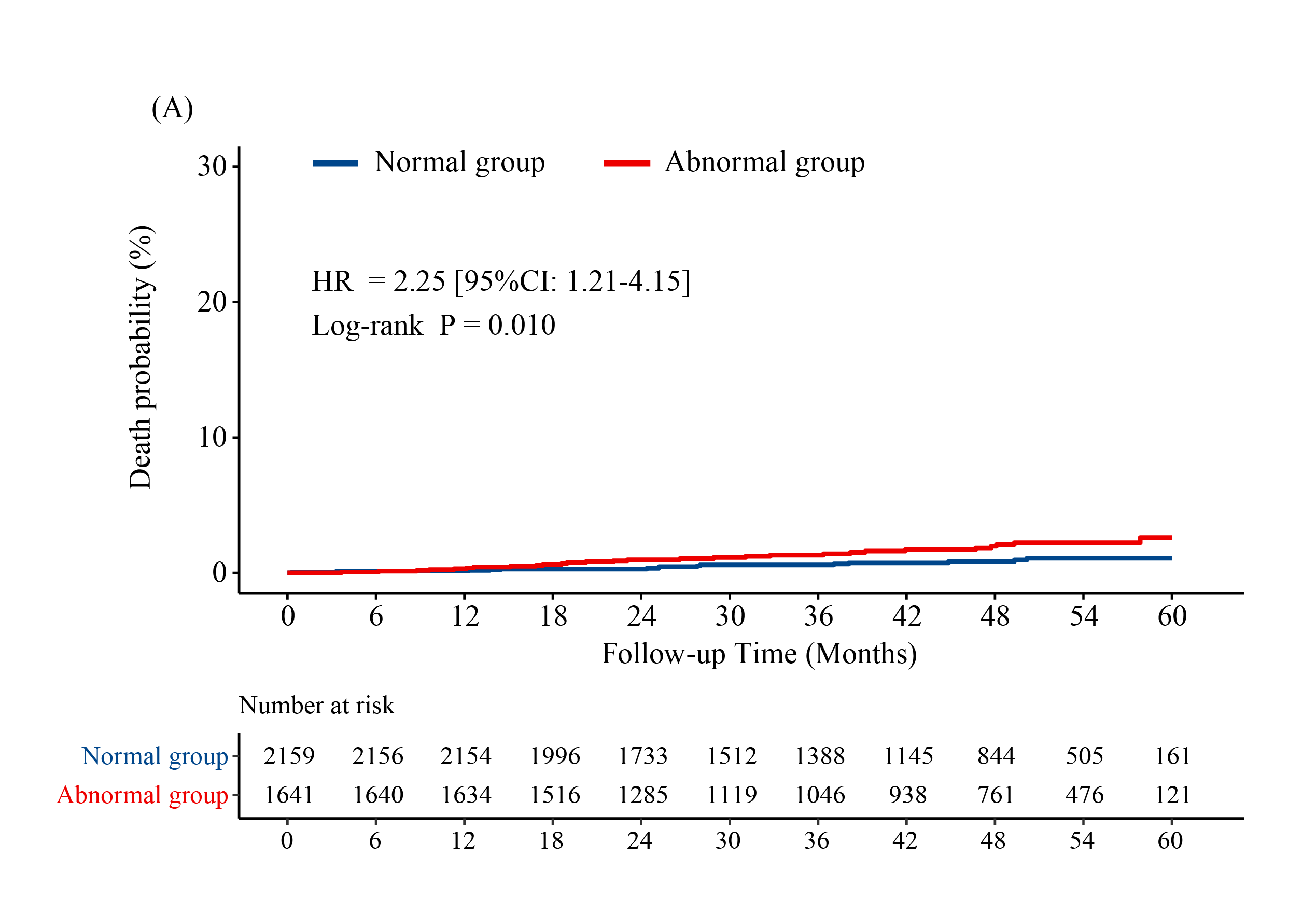** | **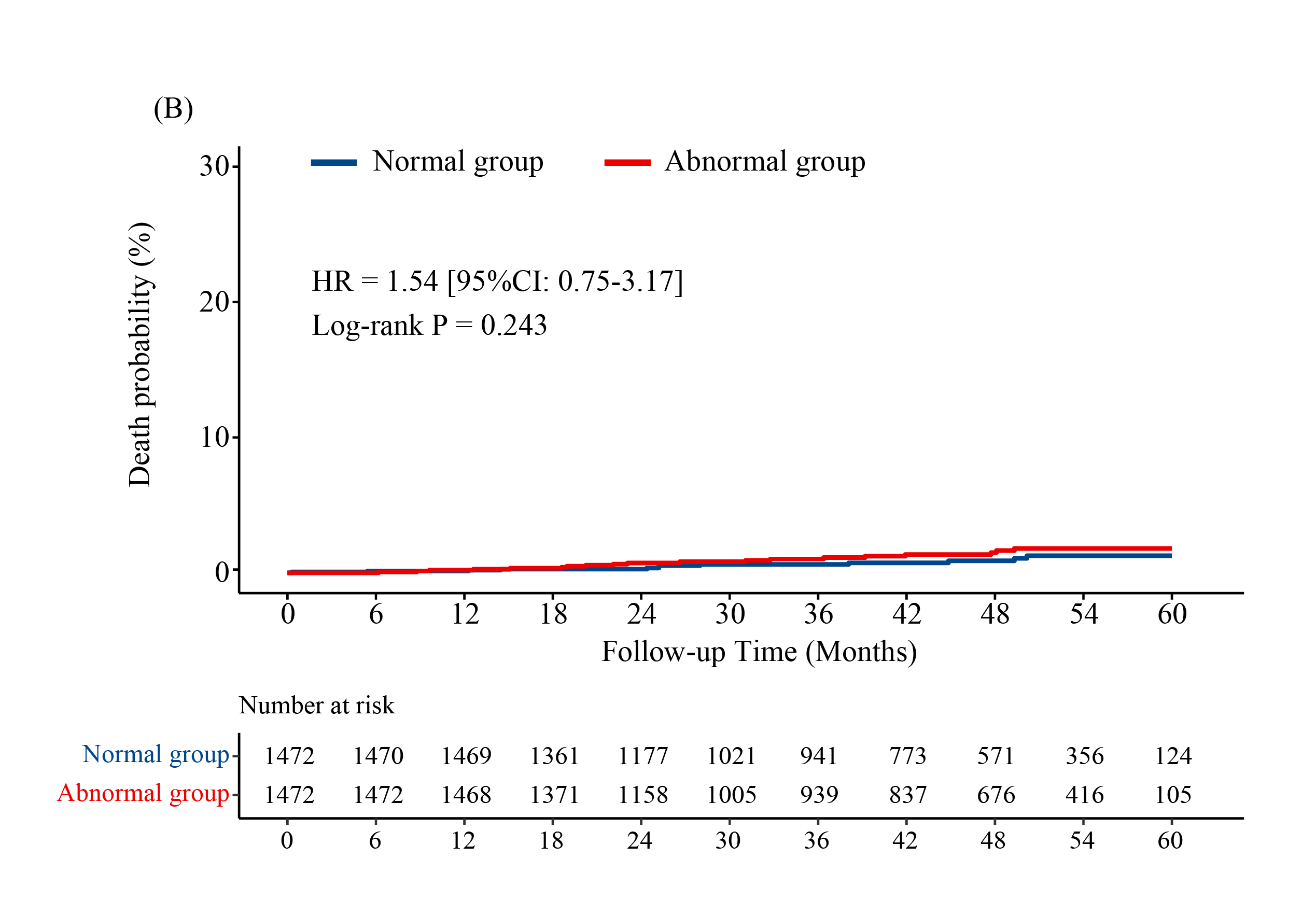** |
| --- | --- |

**Supplementary Fig. 1.** Kaplan-Meier curves for death probability through 5-year follow-up. (A) Before PSM. (B) After PSM.

| **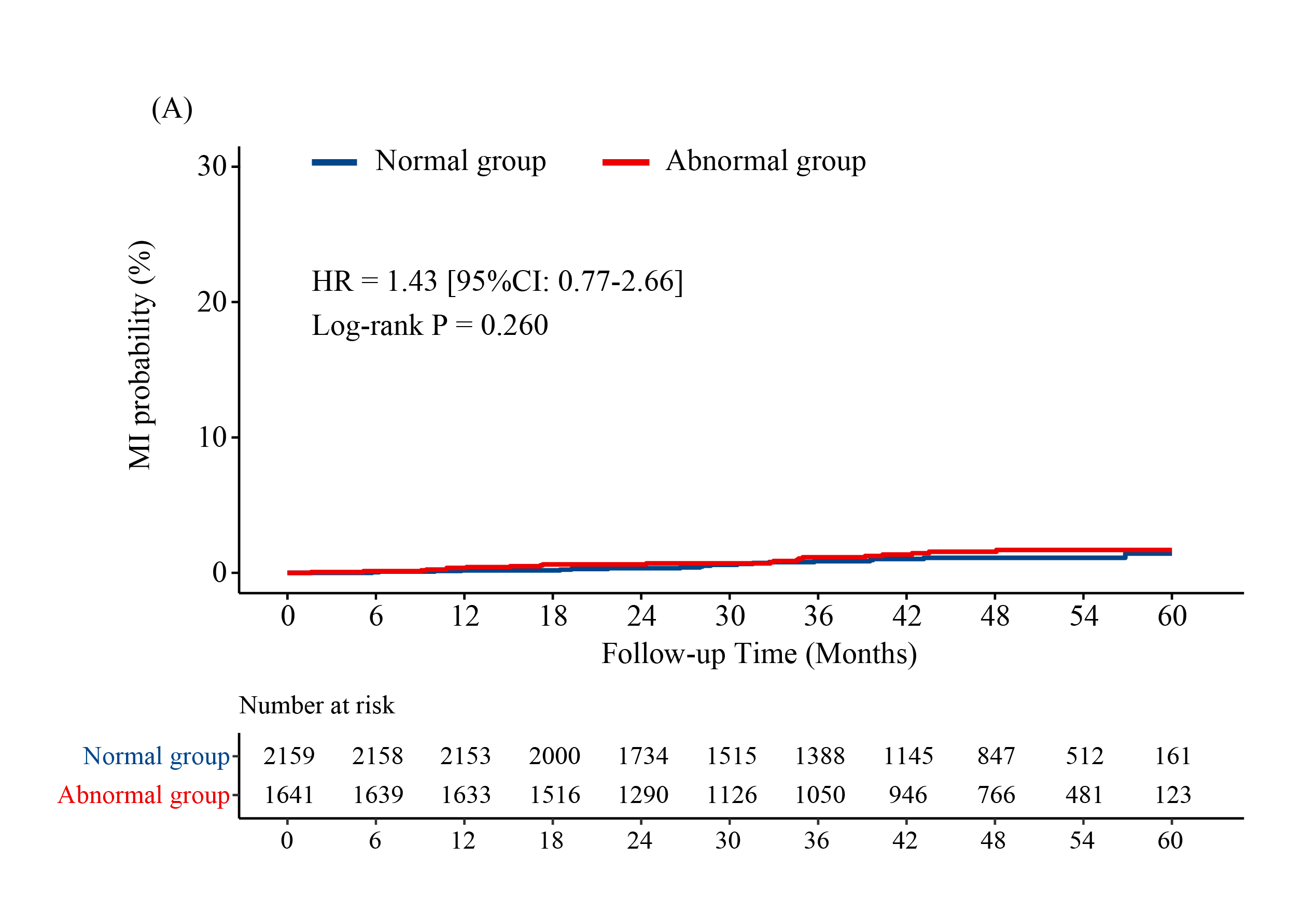** | **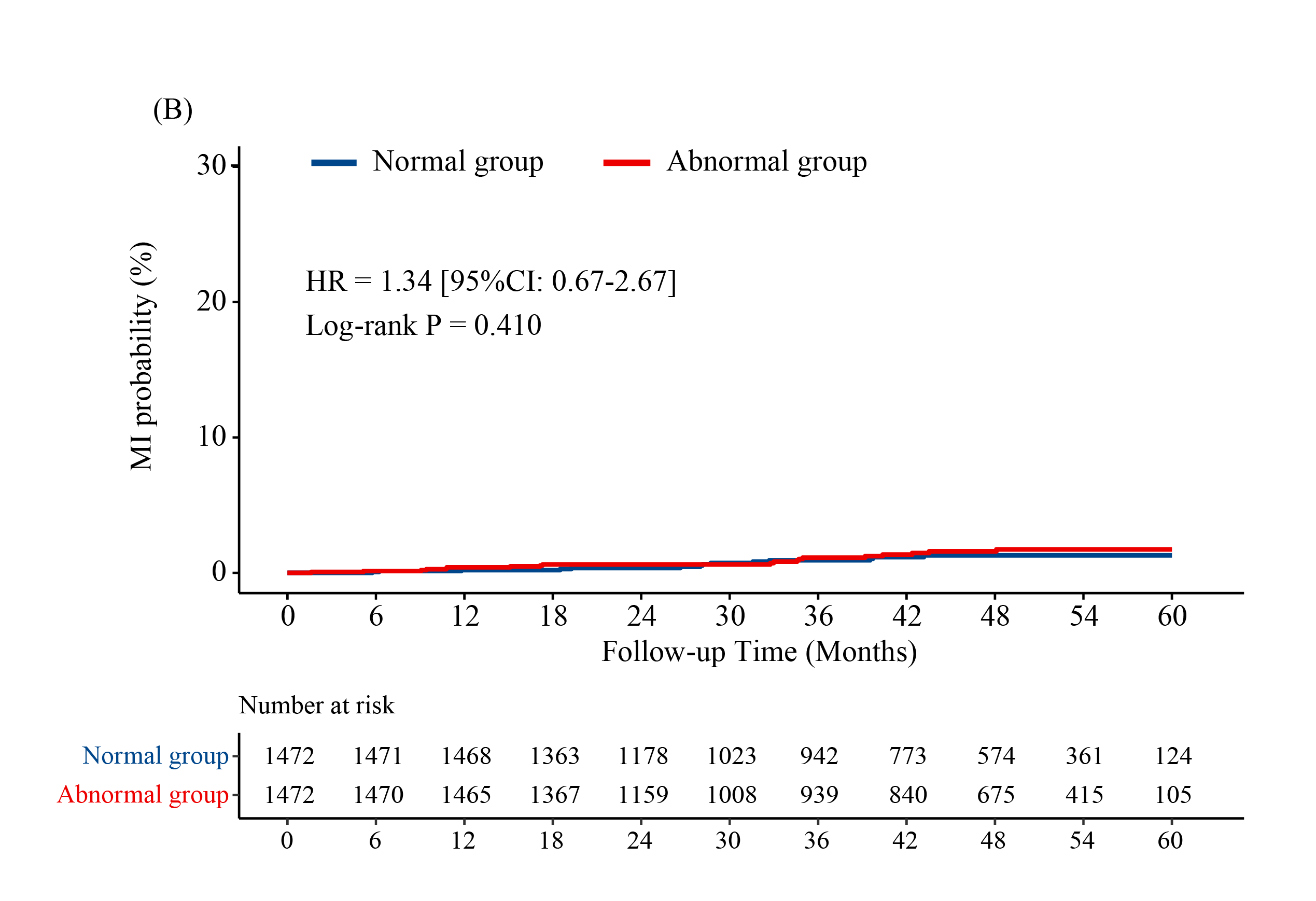** |
| --- | --- |

**Supplementary Fig. 2.** Kaplan-Meier curves for MI probability through 5-year follow-up. (A) Before PSM. (B) After PSM.

| **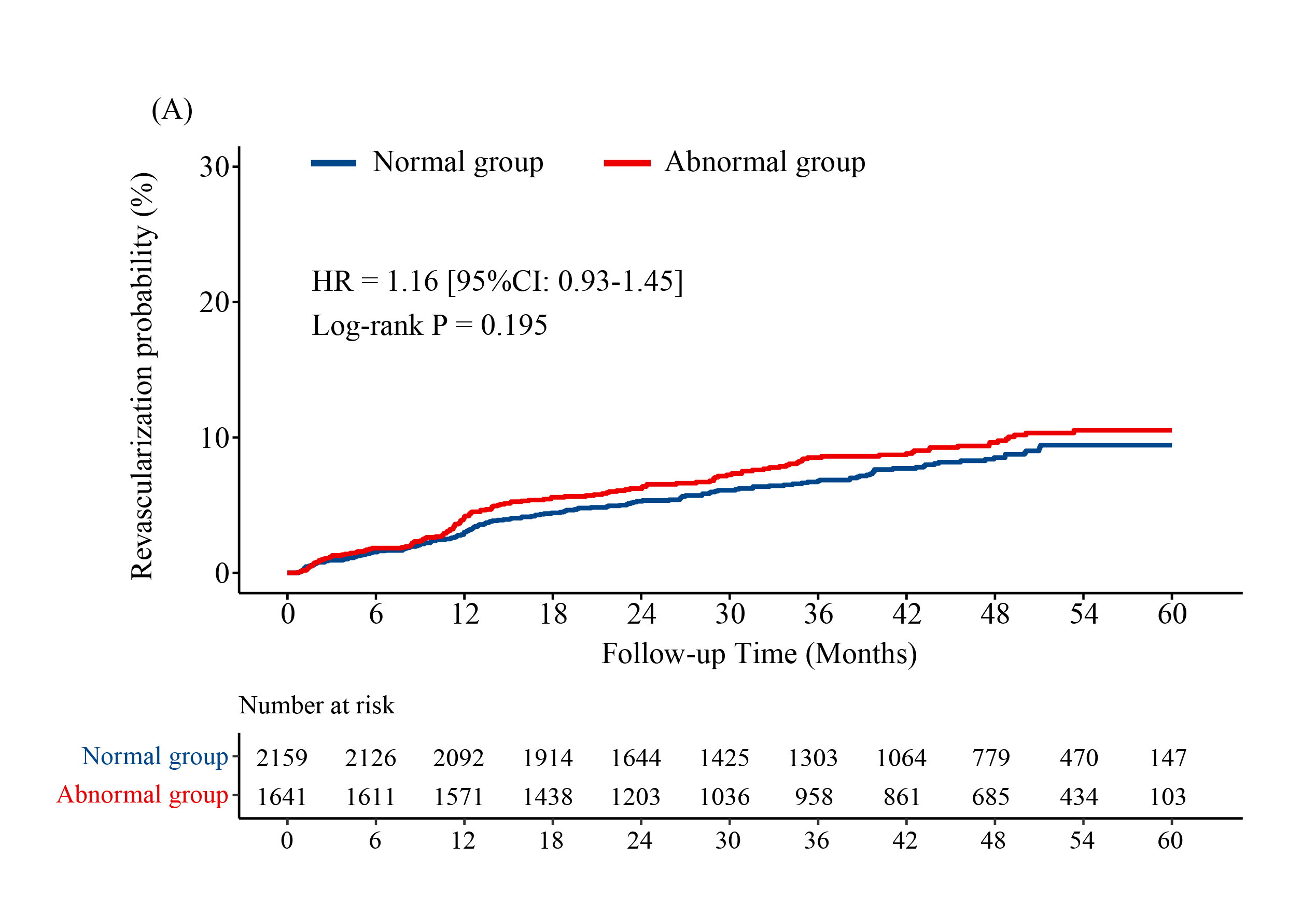** | **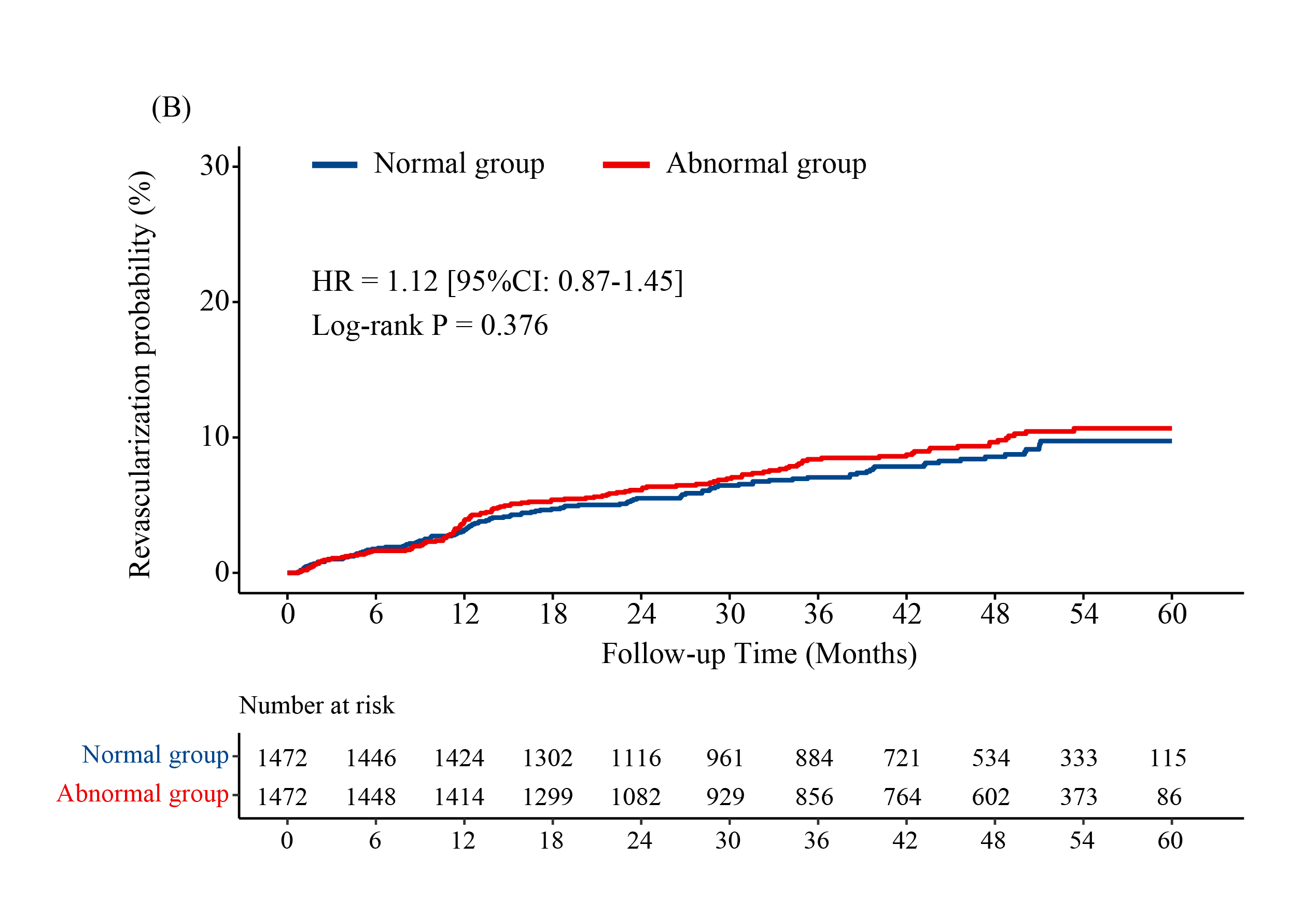** |
| --- | --- |

**Supplementary Fig. 3.** Kaplan-Meier curves for repeated revascularization probability through 5-year follow-up. (A) Before PSM. (B) After PSM.

| **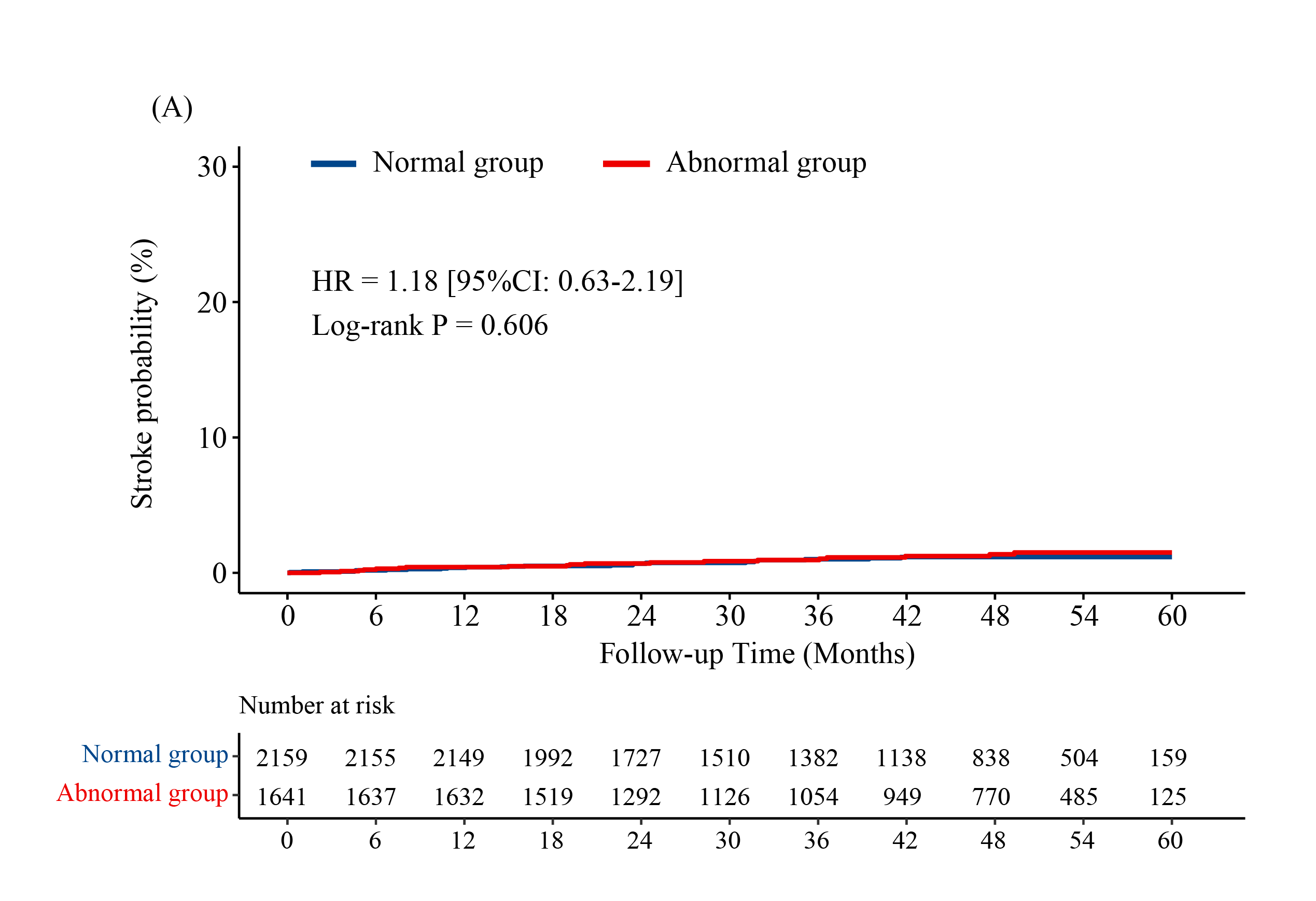** | **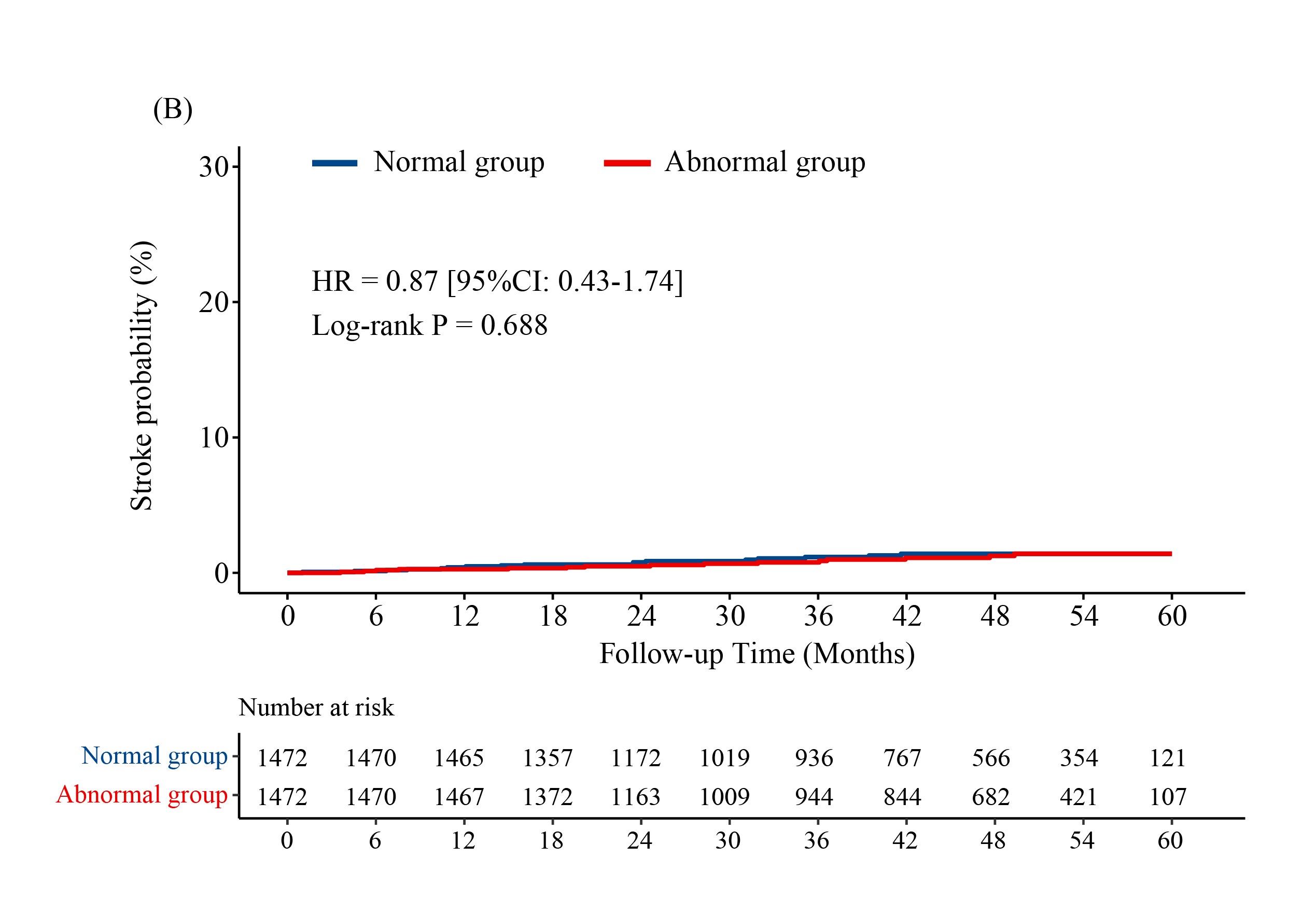** |
| --- | --- |

**Supplementary Fig. 4.** Kaplan-Meier curves for stroke probability through 5-year follow-up. (A) Before PSM. (B) After PSM.

| **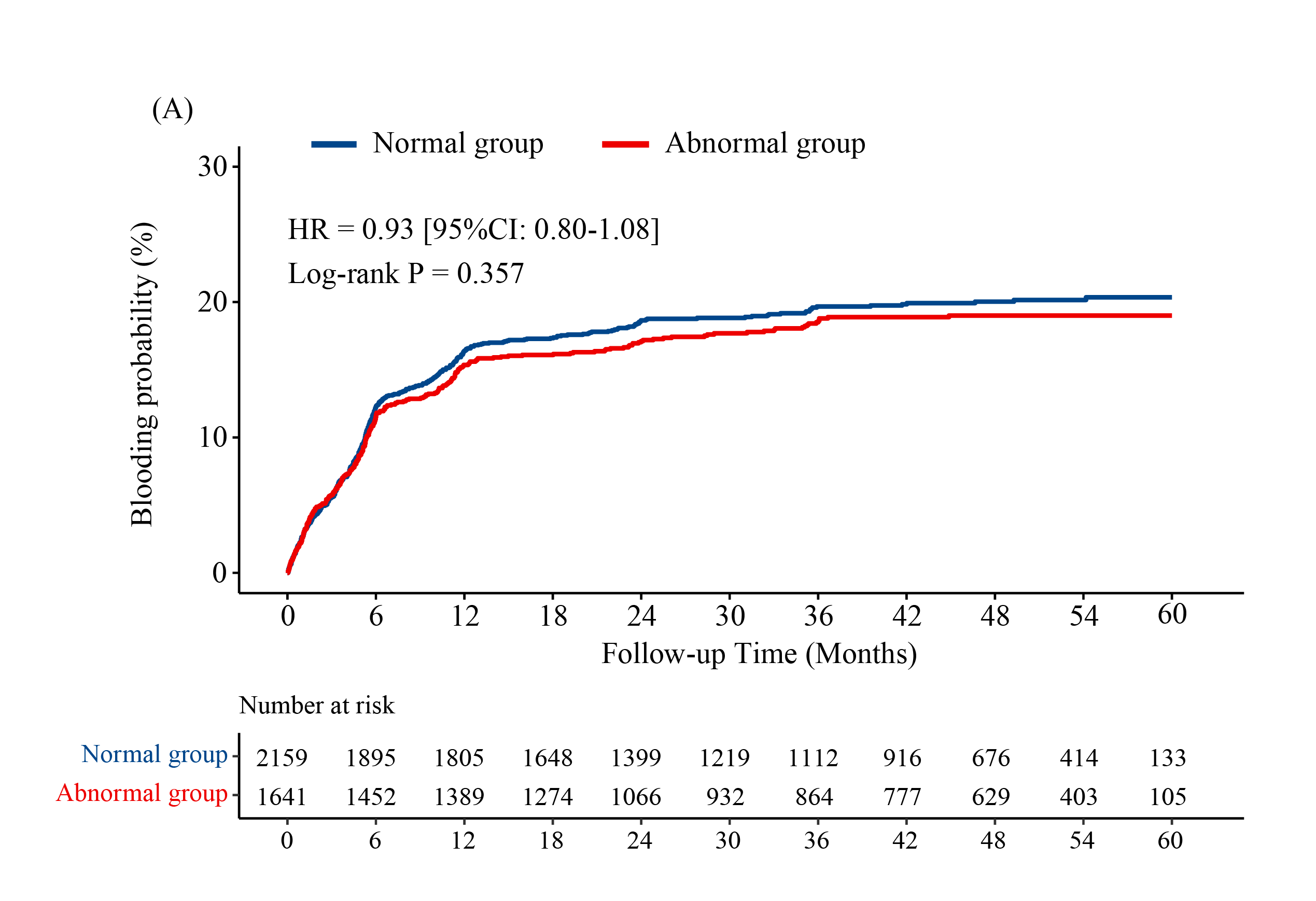** | **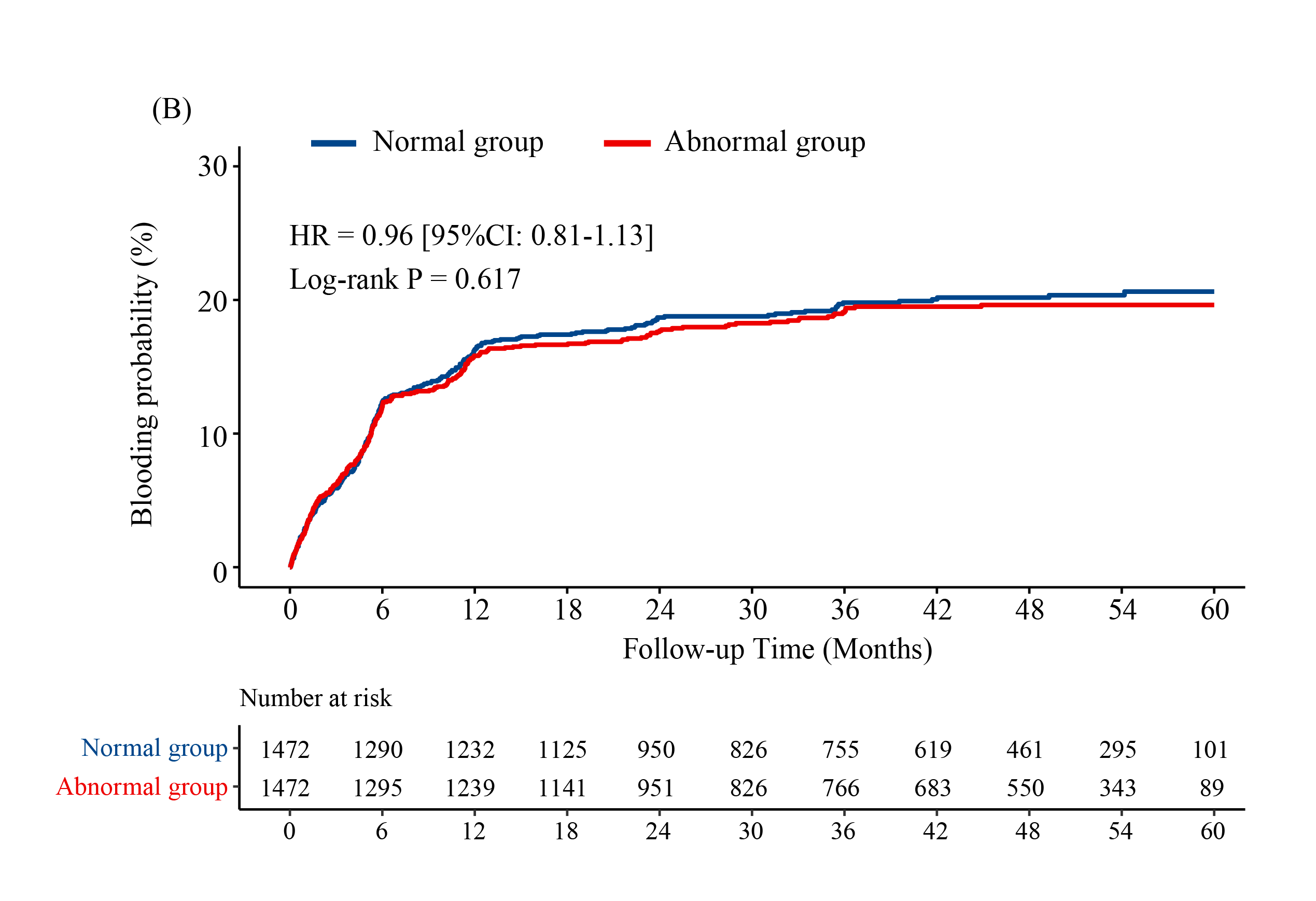** |
| --- | --- |

**Supplementary Fig. 5.** Kaplan-Meier curves for bleeding probability through 5-year follow-up. (A) Before PSM. (B) After PSM.

| **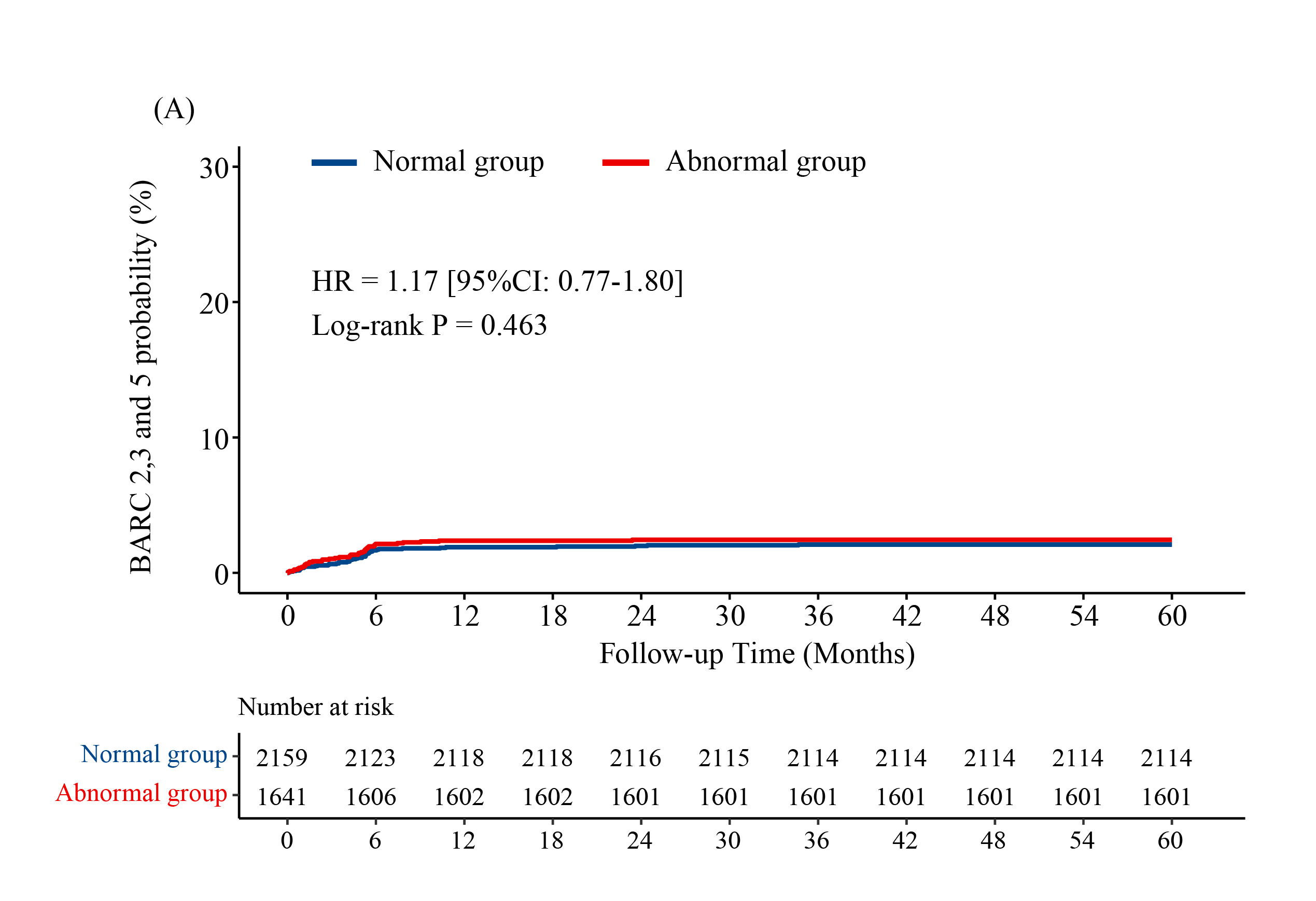** | **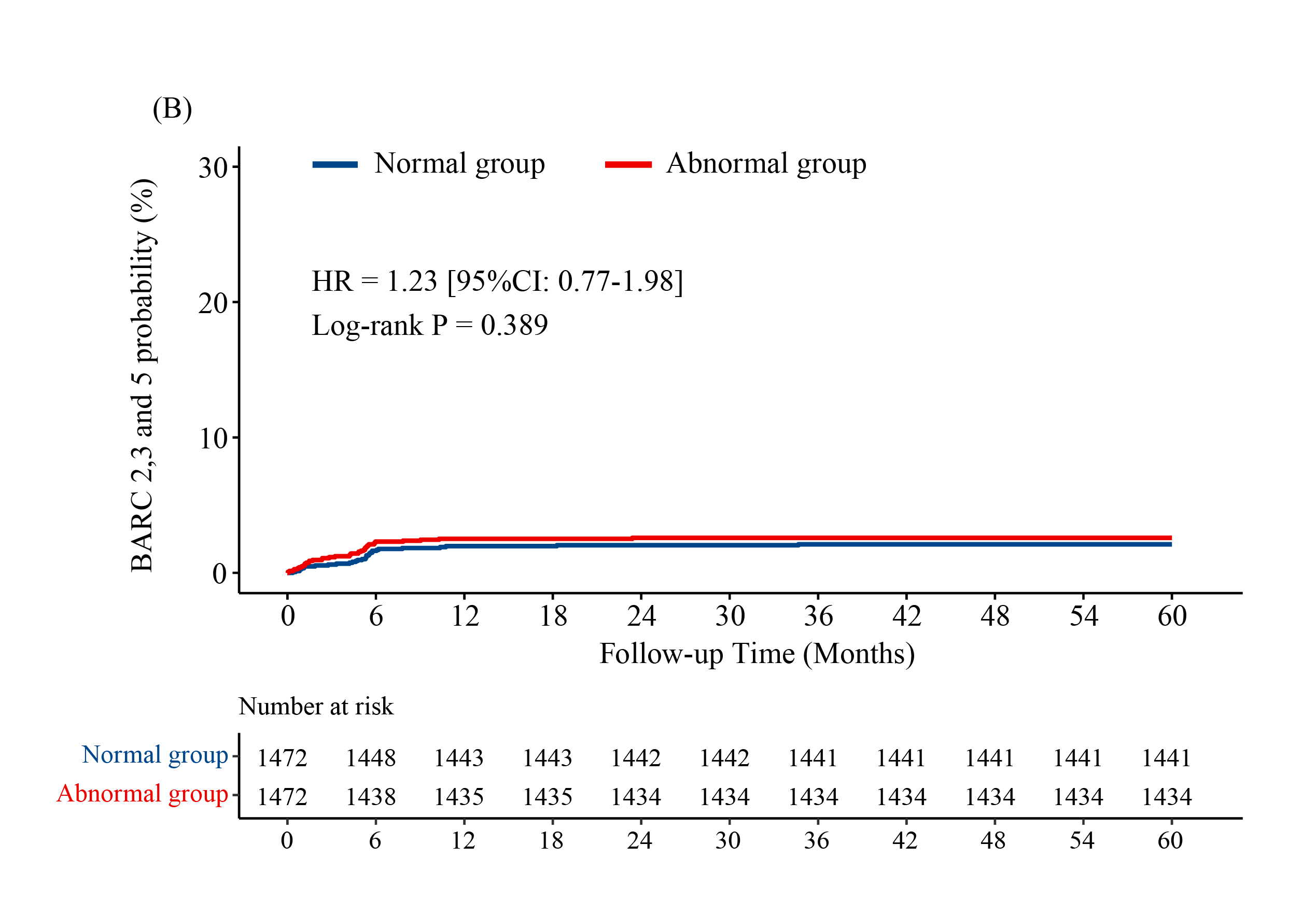** |
| --- | --- |

**Supplementary Fig. 6.** Kaplan-Meier curves for BARC 2, 3 and 5 probability through 5-year follow-up. (A) Before PSM. (B) After PSM.

| **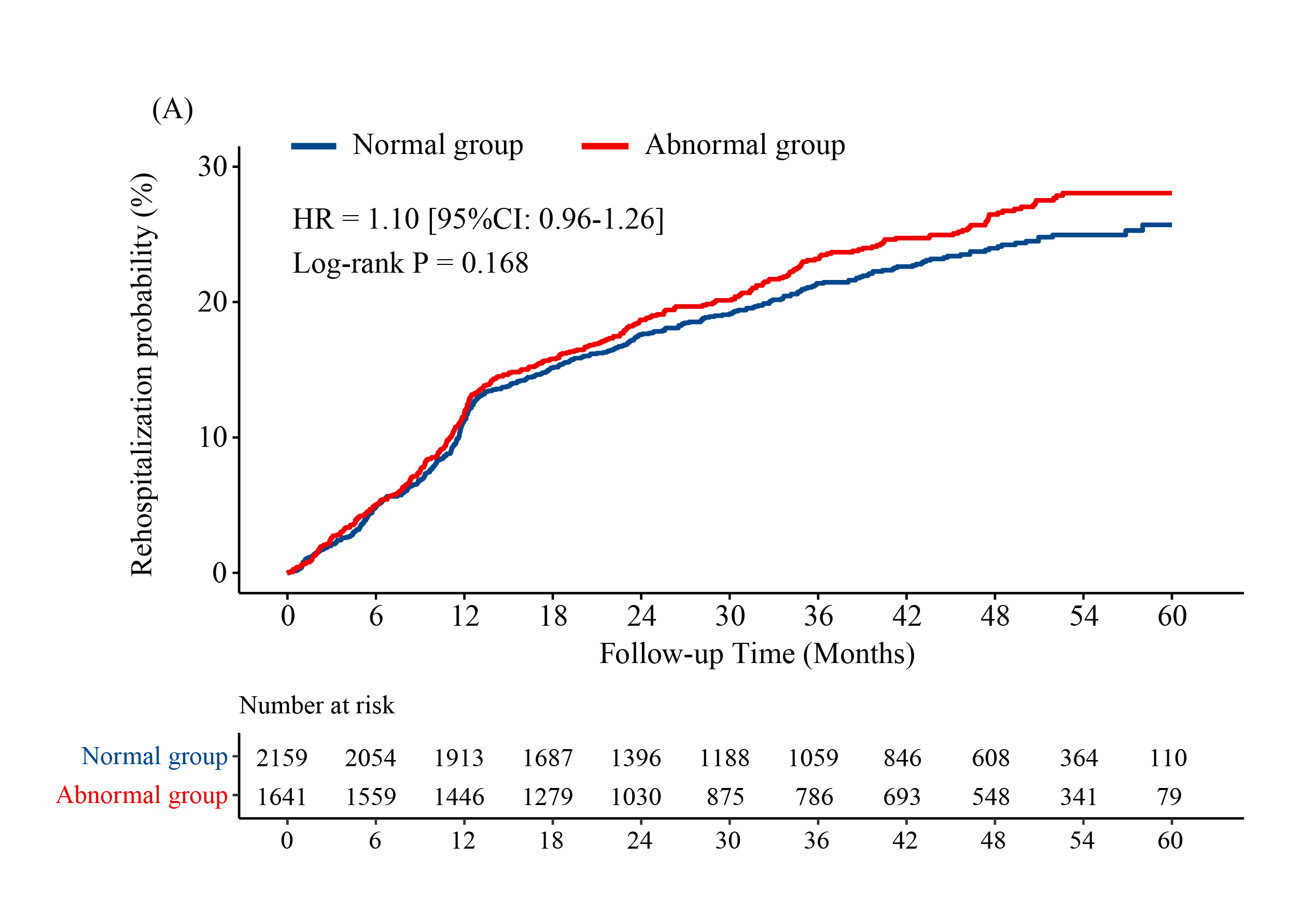** | **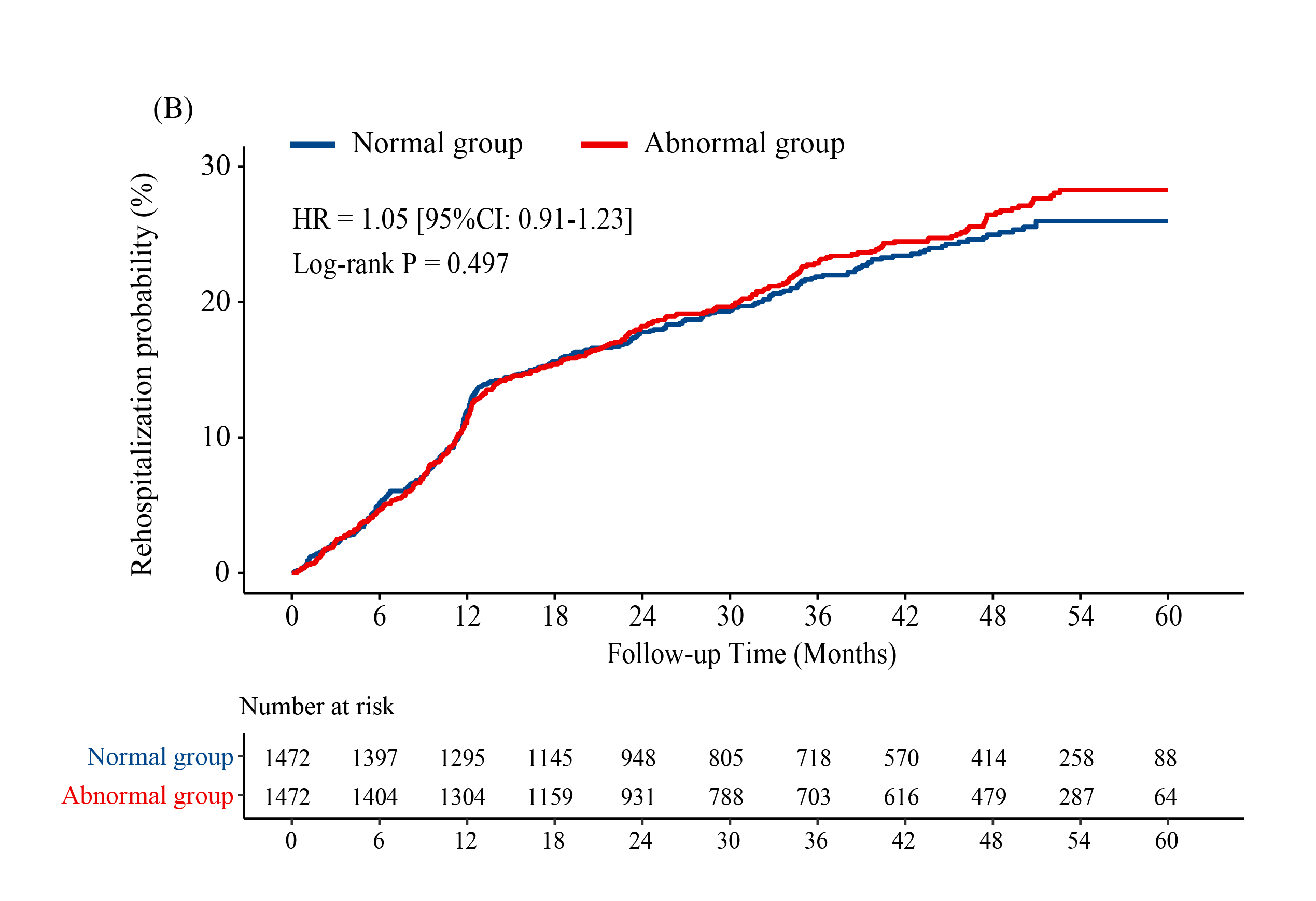** |
| --- | --- |

**Supplementary Fig. 7.** Kaplan-Meier curves for rehospitalization probability through 5-year follow-up. (A) Before PSM. (B) After PSM.

**Supplementary Table 1. Comparison of baseline characteristics before and after PSM in different type of abnormal pulmonary ventilation function**

| Characteristic | Before PSM | | | | After PSM | | | |
| --- | --- | --- | --- | --- | --- | --- | --- | --- |
|  | Obstructive pulmonary  ventilation dysfunction  (N = 260) | Restricted pulmonary  ventilation dysfunction  (N = 1,233) | Mixed pulmonary  ventilation dysfunction  (N = 148) | p-value | Obstructive pulmonary  ventilation dysfunction  (N = 240) | Restricted pulmonary  Ventilation dysfunction  (N = 1,104) | Mixed pulmonary  ventilation dysfunction  (N = 128) | p-value |
| Age (years) | 59.03±8.35 | 58.04±8.77 | 61.27±8.05 | <0.001 | 58.73 ± 8.51 | 57.29 ± 8.62 | 60.39 ± 8.19 | <0.001 |
| Male, no. (%) | 200（76.92%） | 995（80.70%） | 123（83.11%） | 0.250 | 181 (75.42%) | 875 (79.26%) | 103 (80.47%) | 0.370 |
| Current smoking, no. (%) | 110（42.31%） | 500 (40.55%) | 69 (46.62%) | 0.350 | 99 (41.25%) | 427 (38.68%) | 56 (43.75%) | 0.450 |
| Current drinking, no. (%) | 19 (7.31%) | 101 (8.19%) | 15 (10.14%) | 0.600 | 17 (7.08%) | 87 (7.88%) | 12 (9.38%) | 0.740 |
| Past medical history, no. (%) |  |  |  |  |  |  |  |  |
| Hypertension | 149 (57.31%) | 786 (63.75%) | 83 (56.08%) | 0.044 | 137 (57.08%) | 687 (62.23%) | 71 (55.47%) | 0.140 |
| DM | 63 (24.23%) | 385 (31.22%) | 43 (29.05%) | 0.079 | 55 (22.92%) | 334 (30.25%) | 34 (26.56%) | 0.064 |
| CKD | 1 (0.38%) | 4 (0.32%) | 0 (0.00%) | 0.770 | 1 (0.42%) | 3 (0.27%) | 0 (0.00%) | 0.770 |
| Previous PCI | 68 (26.15%) | 375 (30.41%) | 48 (32.43%) | 0.310 | 64 (26.67%) | 347 (31.43%) | 42 (32.81%) | 0.310 |
| Previous stroke | 28 (10.77%) | 134 (10.87%) | 14 (9.46%) | 0.870 | 27 (11.25%) | 106 (9.60%) | 8 (6.25%) | 0.300 |
| Previous MI | 37 (14.23%) | 227 (18.41%) | 26 (17.57%) | 0.280 | 33 (13.75%) | 212 (19.20%) | 23 (17.97%) | 0.140 |
| BMI (kg/m^2^) | 24.95 ± 2.94 | 25.60 ± 2.85 | 25.01 ± 2.73 | <0.001 | 24.94 ± 2.96 | 25.64 ± 2.91 | 25.03 ± 2.78 | <0.001 |
| Heart rate (beats/min) | 74.36 ± 11.62 | 76.56 ± 11.79 | 76.57 ± 12.04 | 0.022 | 74.53 ± 11.61 | 76.46 ± 11.59 | 75.73 ± 11.21 | 0.060 |
| SBP (mmHg) | 136.55 ± 17.32 | 137.79 ± 18.45 | 138.51 ± 17.66 | 0.510 | 136.13 ± 16.94 | 136.90 ± 18.11 | 138.61 ± 17.12 | 0.440 |
| DBP (mmHg) | 80.82 ± 10.03 | 81.08 ± 11.82 | 79.84 ± 10.82 | 0.460 | 80.70 ± 9.89 | 81.05 ± 11.67 | 80.69 ± 10.63 | 0.880 |
| LVEF (%) | 61.05 ± 6.01 | 60.98 ± 6.04 | 61.78 ± 5.42 | 0.300 | 61.37 ± 5.71 | 61.22 ± 5.98 | 61.90 ± 5.53 | 0.460 |
| Diagnosis, no. (%) |  |  |  | 0.320 |  |  |  | 0.130 |
| UA | 225 (86.54%) | 995 (80.70%) | 125 (84.46%) |  | 211 (87.92%) | 897 (81.25%) | 110 (85.94%) |  |
| NSTEMI | 22 (8.46%) | 127 (10.30%) | 11 (7.43%) |  | 21 (8.75%) | 113 (10.24%) | 9 (7.03%) |  |
| STEMI | 13 (5.00%) | 110 (8.92%) | 12 (8.11%) |  | 8 (3.33%) | 93 (8.42%) | 9 (7.03%) |  |
| Medication at Discharge, no. (%) |  |  |  |  |  |  |  |  |
| Aspirin | 256 (98.46%) | 1,223 (99.19%) | 147 (99.32%) | 0.510 | 239 (99.58%) | 1,098 (99.46%) | 128 (100.00%) | 0.690 |
| Clopidogrel | 204 (78.46%) | 992 (80.45%) | 120 (81.08%) | 0.730 | 189 (78.75%) | 885 (80.16%) | 103 (80.47%) | 0.870 |
| Ticagrelor | 85 (32.69%) | 402 (32.60%) | 51 (34.46%) | 0.900 | 75 (31.25%) | 361 (32.70%) | 39 (30.47%) | 0.820 |
| Statin | 254 (97.69%) | 1,206 (97.81%) | 148 (100.00%) | 0.190 | 236 (98.33%) | 1,085 (98.28%) | 128 (100.00%) | 0.330 |
| β-Blocker | 145 (55.77%) | 797 (64.64%) | 78 (52.70%) | 0.001 | 137 (57.08%) | 709 (64.22%) | 67 (52.34%) | 0.007 |
| CCB | 60 (23.08%) | 365 (29.60%) | 42 (28.38%) | 0.110 | 57 (23.75%) | 331 (29.98%) | 38 (29.69%) | 0.150 |
| Nitrates | 159 (61.15%) | 810 (65.69%) | 86 (58.11%) | 0.100 | 147 (61.25%) | 722 (65.40%) | 74 (57.81%) | 0.150 |
| PPI | 118 (45.38%) | 583 (47.28%) | 65 (43.92%) | 0.670 | 108 (45.00%) | 516 (46.74%) | 53 (41.41%) | 0.490 |
| ACEI | 49 (18.85%) | 331 (26.85%) | 37 (25.00%) | 0.026 | 43 (17.92%) | 284 (25.72%) | 33 (25.78%) | 0.036 |
| ARB | 70 (26.92%) | 390 (31.63%) | 43 (29.05%) | 0.300 | 67 (27.92%) | 344 (31.16%) | 34 (26.56%) | 0.390 |
| Diuretic | 10 (3.85%) | 53 (4.30%) | 8 (5.41%) | 0.750 | 8 (3.33%) | 41 (3.71%) | 7 (5.47%) | 0.570 |

Note: DM: diabetes mellitus; CKD: chronic kidney disease; PCI: percutaneous coronary intervention; MI: myocardial infarction; BMI: body mass index; SBP: systolic blood pressure; DBP: diastolic blood pressure; LVEF: left ventricular ejection fraction; UA: unstable angina; NSTEMI: Non-ST segment elevation myocardial infarction; STEMI: ST segment elevation myocardial infarction; CCB: calcium channel blockers; PPI: proton-pump inhibitor; ACEI: angiotensin converting enzyme inhibitors; ARB: angiotensin receptor blocker.

**Supplementary Table 2. Comparison of procedural information and laboratory indicators before and after PSM in different type of abnormal pulmonary ventilation function**

| Characteristic | Before PSM | | | | After PSM | | | |
| --- | --- | --- | --- | --- | --- | --- | --- | --- |
|  | Obstructive pulmonary  ventilation dysfunction  (N = 260) | Restricted pulmonary  ventilation dysfunction  (N = 1,233) | Mixed pulmonary  ventilation dysfunction  (N = 148) | p-value | Obstructive pulmonary  ventilation dysfunction  (N = 240) | Restricted pulmonary  Ventilation dysfunction  (N = 1,104) | Mixed pulmonary  ventilation dysfunction  (N = 128) | p-value |
| Procedural information |  |  |  |  |  |  |  |  |
| SYNTAX score | 10.00 (5.75~15.00) | 10.00 (6.00~16.00) | 10.00 (5.00~16.25) | 0.170 | 10.00 (6.00~15.00) | 10.00 (6.00~15.00) | 9.25 (5.00~15.00) | 0.510 |
| Three-vessel disease, no. (%) | 47 (18.08%) | 192 (15.57%) | 21 (14.19%) | 0.510 | 44 (18.33%) | 171 (15.49%) | 14 (10.94%) | 0.170 |
| PCI, no. (%) | 182 (70.00%) | 927 (75.18%) | 109 (73.65%) | 0.220 | 168 (70.00%) | 824 (74.64%) | 92 (71.88%) | 0.300 |
| CTO, no. (%) | 13 (5.00%) | 36 (2.92%) | 6 (4.05%) | 0.210 | 13 (5.42%) | 31 (2.81%) | 6 (4.69%) | 0.091 |
| Laboratory parameters |  |  |  |  |  |  |  |  |
| Hemoglobin (g/L) | 138.51 ± 12.82 | 140.29 ± 14.11 | 140.29 ± 14.06 | 0.170 | 138.71 ± 12.92 | 140.47 ± 14.12 | 140.09 ± 14.16 | 0.210 |
| NT-PorBNP (pg/ml) | 72.73 (33.99~164.93) | 81.34 (36.97~221.50) | 81.82 (43.73~202.08) | 0.690 | 71.92 (33.70~160.05) | 79.08 (35.12~204.15) | 80.10 (43.22~185.20) | 0.500 |
| Total cholesterol (mmol/L) | 3.67 (3.03~4.42) | 3.73 (3.11~4.45) | 3.82 (3.17~4.54) | 0.280 | 3.71 (3.06~4.44) | 3.70 (3.09~4.41) | 3.79 (3.17~4.54) | 0.330 |
| Triglycerides (mmol/L) | 1.36 (0.96~1.95) | 1.45 (1.03~1.99) | 1.35 (1.01~1.95) | 0.580 | 1.35 (0.96~1.95) | 1.44 (1.03~1.98) | 1.29 (1.00~1.97) | 0.480 |
| LDL-C (mmol/L) | 1.94 (1.53~2.48) | 1.99 (1.59~2.54) | 2.12 (1.65~2.74) | 0.079 | 1.94 (1.54~2.51) | 1.98 (1.57~2.52) | 2.13 (1.63~2.74) | 0.078 |
| HDL-C (mmol/L) | 1.08 (0.94~1.26) | 1.04 (0.90~1.19) | 1.08 (0.93~1.21) | 0.001 | 1.09 (0.96~1.28) | 1.04 (0.91~1.20) | 1.08 (0.95~1.22) | 0.002 |
| Troponin (ng/mL) | 0.01 (0.01~0.01) | 0.01 (0.01~0.02) | 0.01 (0.01~0.02) | 0.160 | 0.01 (0.01~0.01) | 0.01 (0.01~0.02) | 0.01 (0.01~0.02) | 0.130 |
| Scr (μmol / L) | 70.24 (61.85~79.88) | 69.45 (59.53~79.12) | 69.26 (61.36~76.34) | 0.350 | 70.32 (61.89~79.76) | 69.22 (59.08~78.73) | 69.19 (61.42~76.23) | 0.310 |
| ALT (U/L) | 20.33 (15.00~29.93) | 23.00 (16.73~35.62) | 20.56 (15.00~31.00) | 0.120 | 20.50 (15.00~29.93) | 23.13 (17.00~35.79) | 20.56 (15.00~31.22) | 0.140 |
| CKMB (U/L) | 12.10 (10.00~16.00) | 12.00 (10.00~16.00) | 12.85 (10.00~16.00) | 0.640 | 12.00 (9.65~16.00) | 12.00 (10.00~16.00) | 12.85 (10.00~16.00) | 0.590 |

Note: SYNTAX score: the synergy between percutaneous coronary intervention with taxus and cardiac surgery score; PCI: percutaneous coronary intervention; CTO: chronic total occlusion; NT-PorBNP: N-terminal Pro-B-type natriuretic peptide; LDL-C: low-density lipoprotein cholesterol; HDL-C: high-density lipoprotein cholesterol; Scr: serum creatinine; ALT: alanine aminotransferase; CKMB: Creatine kinase MB.

**Supplementary Table 3. Comparison CPET parameters before and after PSM in different type of abnormal pulmonary ventilation function**

| Characteristic | Before PSM | | | | After PSM | | | |
| --- | --- | --- | --- | --- | --- | --- | --- | --- |
|  | Obstructive pulmonary  ventilation dysfunction  (N = 260) | Restricted pulmonary  ventilation dysfunction  (N = 1,233) | Mixed pulmonary  ventilation dysfunction  (N = 148) | p-value | Obstructive pulmonary  ventilation dysfunction  (N = 240) | Restricted pulmonary  Ventilation dysfunction  (N = 1,104) | Mixed pulmonary  ventilation dysfunction  (N = 128) | p-value |
| Static lung function |  |  |  |  |  |  |  |  |
| FVC (L/min) | 3.43 ± 0.65 | 2.64 ± 0.58 | 2.58 ± 0.62 | <0.001 | 3.42 ± 0.67 | 2.65 ± 0.58 | 2.57 ± 0.62 | <0.001 |
| FVC (% predicted) | 87.68 ± 6.18 | 69.89 ± 8.24 | 69.57 ± 10.56 | <0.001 | 87.78 ± 6.29 | 70.06 ± 8.16 | 69.41 ± 9.98 | <0.001 |
| FEV_1_ (L) | 2.13 ± 0.50 | 2.24 ± 0.50 | 1.57 ± 0.44 | <0.001 | 2.13 ± 0.50 | 2.25 ± 0.51 | 1.57 ± 0.44 | <0.001 |
| FEV_1_ (% predicted) | 71.12 ± 12.13 | 73.39 ± 9.78 | 54.01 ± 11.88 | <0.001 | 71.15 ± 11.97 | 73.50 ± 9.66 | 53.65 ± 12.04 | <0.001 |
| FEV_1_/FVC (%) | 62.04 ± 8.28 | 85.19 ± 10.58 | 60.58 ± 7.16 | <0.001 | 62.08 ± 8.15 | 85.24 ± 10.27 | 60.83 ± 7.02 | <0.001 |
| FEV_1_/FVC (% predicted) | 80.64 ± 10.76 | 82.88 ± 7.30 | 79.17 ± 9.39 | <0.001 | 80.61 ± 10.62 | 83.00 ± 7.30 | 79.35 ± 9.30 | <0.001 |
| VCmax (L/min) | 3.55 ± 0.65 | 2.89 ± 0.61 | 2.84 ± 0.64 | <0.001 | 3.54 ± 0.67 | 2.90 ± 0.62 | 2.84 ± 0.63 | <0.001 |
| MVV (L/min) | 99.73 ± 24.38 | 95.40 ± 25.75 | 75.50 ± 24.52 | <0.001 | 99.84 ± 24.32 | 96.09 ± 25.86 | 75.23 ± 25.44 | <0.001 |
| Dynamic lung function |  |  |  |  |  |  |  |  |
| Duration (seconds) | 392.19 ± 110.86 | 389.08 ± 106.47 | 384.07 ± 102.96 | 0.760 | 391.63 ± 110.90 | 390.77 ± 107.15 | 383.54 ± 104.06 | 0.750 |
| Peak heart rate (time/min) | 117.22 ± 18.98 | 117.02 ± 18.85 | 114.82 ± 16.10 | 0.380 | 117.65 ± 19.03 | 117.43 ± 18.93 | 115.42 ± 16.28 | 0.490 |
| Peak workload (W) | 92.53 ± 28.66 | 92.31 ± 27.77 | 88.55 ± 27.95 | 0.290 | 92.34 ± 28.75 | 92.82 ± 28.16 | 88.66 ± 27.84 | 0.290 |
| Peak MET | 4.52 ± 1.15 | 4.21 ± 1.05 | 4.25 ± 0.97 | <0.001 | 4.52 ± 1.14 | 4.24 ± 1.06 | 4.28 ± 0.95 | <0.001 |
| MET AT | 3.14 ± 0.59 | 3.00 ± 0.58 | 3.10 ± 0.56 | 0.002 | 3.13 ± 0.58 | 3.01 ± 0.59 | 3.10 ± 0.56 | 0.005 |
| Peak VO_2_ (ml/kg/min) | 15.81 ± 4.02 | 14.74 ± 3.66 | 14.86 ± 3.41 | <0.001 | 15.84 ± 3.99 | 14.83 ± 3.70 | 14.96 ± 3.32 | <0.001 |
| VO_2_ AT (ml/kg/min) | 10.96 ± 2.07 | 10.50 ± 2.05 | 10.80 ± 1.97 | 0.002 | 10.95 ± 2.04 | 10.52 ± 2.07 | 10.83 ± 1.95 | 0.007 |
| Peak VE (L/min) | 39.45 ± 12.32 | 38.12 ± 11.52 | 37.59 ± 10.70 | 0.180 | 39.31 ± 12.34 | 38.23 ± 11.67 | 37.20 ± 10.42 | 0.230 |
| Peak Oxygen pulse (ml/beat) | 9.72 ± 2.42 | 9.42 ± 2.44 | 9.40 ± 2.23 | 0.190 | 9.69 ± 2.46 | 9.45 ± 2.48 | 9.41 ± 2.24 | 0.350 |
| Maximal respiratory rate (time/min) |  |  |  |  |  |  |  |  |
| VE/VO_2_ | 29.70 ± 3.86 | 29.56 ± 4.01 | 30.80 ± 4.87 | 0.002 | 29.58 ± 3.84 | 29.39 ± 3.98 | 30.27 ± 4.82 | 0.064 |
| VE/VCO_2_ | 30.94 ± 3.82 | 30.50 ± 3.86 | 31.75 ± 4.50 | <0.001 | 30.78 ± 3.78 | 30.31 ± 3.82 | 31.29 ± 4.44 | 0.011 |
| HRR | 43.25 ± 18.06 | 44.42 ± 18.88 | 43.38 ± 15.83 | 0.560 | 43.11 ± 18.14 | 44.78 ± 18.95 | 43.68 ± 15.95 | 0.400 |
| BR (%) | 59.18 ± 12.81 | 58.66 ± 11.98 | 46.77 ± 17.44 | <0.001 | 59.38 ± 12.83 | 58.89 ± 11.90 | 46.41 ± 17.36 | <0.001 |

Note: FVC: forced vital capacity; FEV_1_: forced expiratory volume in one second; VCmax: maximum vital capacity; MVV: maximal voluntary ventilation; MET: metabolic equivalent; AT: anaerobic threshold; VO_2_: oxygen consumption; VE: minute ventilation volume; VE/VO_2_: the minute ventilation-oxygen dioxide production; VE/VCO_2_: the minute ventilation-carbon dioxide production; HRR: heart rate reserve; BR: breathing reserve.

**Supplementary Table 4. Uni- and multivariable Logistic regression analysis for pulmonary ventilation dysfunction**

| **Characteristic** | **Univariable Logistic regression** | | | **Multivariable Logistic regression** | |
| --- | --- | --- | --- | --- | --- |
|  | **OR (95% CI)** | **P-value** | **OR (95% CI)** | | **P-value** |
| Age | 1.03 (1.02-1.04) | <0.001 | 1.04 (1.03-1.04) | | <0.001 |
| Male | 1.42 (1.22-1.66) | <0.001 | 1.43 (1.20-1.71) | | <0.001 |
| Current smoking | 1.22 (1.07-1.40) | 0.003 | 1.20 (1.04-1.38) | | 0.015 |
| Current drinking | 1.10 (0.86-1.39) | 0.443 | - | | - |
| Hypertension | 1.14 (1.00-1.30) | 0.051 | - | | - |
| DM | 1.28 (1.11-1.48) | <0.001 | 1.20 (1.04-1.40) | | 0.014 |
| CKD | 1.65 (0.44-6.66) | 0.456 | - | | - |
| Previous PCI | 0.98 (0.85-1.13) | 0.805 | - | | - |
| Previous stroke | 1.22 (0.98-1.51) | 0.074 | - | | - |
| Previous MI | 0.97 (0.82-1.15) | 0.727 | - | | - |
| BMI | 0.99 (0.97-1.01) | 0.333 | - | | - |
| Heart rate | 1.00 (1.00-1.01) | 0.550 | - | | - |
| SBP | 1.01 (1.00-1.01) | <0.001 | 1.00 (1.00-1.01) | | 0.017 |
| DBP | 1.00 (0.99-1.00) | 0.754 | - | | - |
| LVEF | 0.98 (0.97-0.99) | <0.001 | 0.98 (0.97-0.99) | | 0.003 |
| Diagnosis |  | 0.545 | - | | - |
| UA | Ref |  | - | | - |
| NSTEMI | 1.06 (0.85-1.32) |  | - | | - |
| STEMI | 1.16 (0.91-1.47) |  | - | | - |
| Aspirin | 0.50 (0.22-1.11) | 0.090 | - | | - |
| Clopidogrel | 1.03 (0.88-1.21) | 0.740  0.549 | - | | - |
| Ticagrelor | 1.04 (0.91-1.20) |  | - | | - |
| Statin | 0.64 (0.38-1.06) | 0.085 | - | | - |
| β-Blocker | 1.05 (0.92-1.20) | 0.486 | - | | - |
| CCB | 1.00 (0.87-1.15) | 0.985 | - | | - |
| Nitrates | 1.01 (0.88-1.16) | 0.882 | - | | - |
| PPI | 1.02 (0.90-1.16) | 0.738 | - | | - |
| ACEI | 1.07 (0.92-1.24) | 0.365 | - | | - |
| ARB | 1.03 (0.90-1.18) | 0.672 | - | | - |
| Diuretic | 1.22 (0.88-1.70) | 0.232 | - | | - |
| SYNTAX score | 1.01 (1.00-1.02) | 0.013 | - | | - |
| Three-vessel disease | 1.01 (0.84-1.20) | 0.936 | - | | - |
| PCI | 1.09 (0.94-1.26) | 0.256 | - | | - |
| CTO | 1.03 (0.72-1.48) | 0.852 | - | | - |
| Hemoglobin | 1.00 (0.99-1.00) | 0.266 | - | | - |
| NT-PorBNP | 1.00 (1.00-1.00) | 0.013 | - | | - |
| Total cholesterol | 0.99 (0.93-1.05) | 0.788 | - | | - |
| Triglycerides | 1.06 (1.00-1.11) | 0.050 | - | | - |
| LDL-C | 1.01 (0.92-1.09) | 0.901 | - | | - |
| HDL-C | 0.53 (0.40-0.70) | <0.001 | 0.57 (0.43-0.76) | | <0.001 |
| Troponin | 0.97 (0.83-1.11) | 0.645 | - | | - |
| Scr | 1.00 (1.00-1.01) | 0.244 | - | | - |
| ALT | 1.00 (1.00-1.00) | 0.630 | - | | - |
| CKMB | 1.00 (0.99-1.00) | 0.368 | - | | - |

Note: DM: diabetes mellitus; CKD: chronic kidney disease; PCI: percutaneous coronary intervention; MI: myocardial infarction; BMI: body mass index; SBP: systolic blood pressure; DBP: diastolic blood pressure; LVEF: left ventricular ejection fraction; UA: unstable angina; NSTEMI: Non-ST segment elevation myocardial infarction; STEMI: ST segment elevation myocardial infarction; CCB: calcium channel blockers; PPI: proton-pump inhibitor; ACEI: angiotensin converting enzyme inhibitors; ARB: angiotensin receptor blocker; SYNTAX score: the synergy between percutaneous coronary intervention with taxus and cardiac surgery score; CTO: chronic total occlusion; NT-PorBNP: N-terminal Pro-B-type natriuretic peptide; LDL-C: low-density lipoprotein cholesterol; HDL-C: high-density lipoprotein cholesterol; Scr: serum creatinine; ALT: alanine aminotransferase; CKMB: Creatine kinase MB.
